# Surface functional groups and degree of carbonization of selected chars from different processes and feedstock

**DOI:** 10.1371/journal.pone.0277365

**Published:** 2022-11-17

**Authors:** Marija Ilić, Franz-Hubert Haegel, Aleksandar Lolić, Zoran Nedić, Tomislav Tosti, Ivana Sredović Ignjatović, Andreas Linden, Nicolai D. Jablonowski, Heinrich Hartmann

**Affiliations:** 1 Faculty of Mining and Geology, University of Belgrade, Belgrade, Serbia; 2 Institute of Bio- und Geosciences – Agrosphere (IBG-3), Forschungszentrum Jülich GmbH, Jülich, Germany; 3 Faculty of Chemistry, University of Belgrade, Belgrade, Serbia; 4 Faculty of Physical Chemistry, University of Belgrade, Belgrade, Serbia; 5 Faculty of Agriculture, University of Belgrade, Belgrade, Serbia; 6 Institute of Bio- und Geosciences – Plant Sciences (IBG-2), Forschungszentrum Jülich GmbH, Jülich, Germany; 7 Central Institute for Engineering, Electronics and Analytics – Analytics (ZEA-3), Forschungszentrum Jülich GmbH, Jülich, Germany; Universiti Teknologi Petronas: Universiti Teknologi PETRONAS, MALAYSIA

## Abstract

The knowledge of the structural and chemical properties of biochars is decisive for their application as technical products. For this reason, methods for the characterization of biochars that are generally applicable and allow quality control are highly desired. Several methods that have shown potential in other studies were used to investigate two activated carbons and seven biochars from different processes and feedstock. The chars were chosen to cover a wide range of chemical composition and structural properties as a hardness test for the analytical methods used in this study. Specific problems connected with the pretreatment of samples and drawbacks of some methods for some types of chars could be identified in an integrated consideration of the results from different methods. None of the spectroscopic methods was found to be suitable for the quality control of all types of chars. The most valuable results were obtained by chemical analysis that, however, required the complete determination of the main elements, including that of oxygen, and of inorganic components for adequate results. The combination of X-ray photoelectron spectroscopy (XPS) and FT-IR spectroscopy allows a rough characterization of surface functional groups, but cannot discriminate aliphatic and aromatic OH groups. FT-IR might be a suitable method for the quality control of biochars made at lower temperature. The results of Raman spectroscopy did not well correlate with the amount of sp^2^ hybridized carbon determined by XPS. A better correlation of XPS data was found with the electrical polarization determined by the method of spectral induced polarization that was used for the first time in conjunction with extensive analytical characterization.

## Introduction

Carbon materials play an important role in technical products and processes [1,2] as well as in the environment [3–6]. Whereas the technical application of carbon black and activated carbon has long tradition also including activated carbons from biomass [7], the large-scale carbonization of biomass has found major interest in the last decade as a source for bioenergy [8], for the production of a valuable carbonaceous material (biochar) and as a means for carbon sequestration [9,10]. Meanwhile thousands of articles are available on the production, use and modification of biochars as a sustainable carbon source. Two main types of production processes are used to carbonize biomass either in contact with a gas phase (pyrolysis or gasification) or in the presence of liquid water under pressure (hydrothermal carbonization). Pyrolysis is typically performed under the complete exclusion of oxygen [11] in a temperature range between 350 and 800 °C, whereas gasification usually takes place in the presence of oxygen, steam or carbon dioxide at typical temperatures of 800 °C or more [10,12]. The typical temperature range for hydrothermal carbonization is 150–300 °C [13]. Many different ways of technical realizations exist for all of the processes mentioned above, including the use of microwave [14,15] and solar heating [16]. Some of the processes are optimized for the production of liquid and gaseous fuels with biochar as a by-product [17], others are optimized for producing designed chars.

Biochar is a suitable basic material for the production of activated carbon [18–21]. Sometimes it can also favorably substitute activated carbon with no or inexpensive modification due to lower costs [22,23] or improved properties [22]. It may show comparable or even better performance than classical carbon materials when used as adsorbent [22,24], electrode material [25,26], catalyst [27–29], catalyst support [30], filler in polymers [31,32] and building materials [33,34] or component in other composites [35].

The most discussed application of biochar is their use as a soil amendment leading to improved crop yield [36] and sequestration of a large amount of carbon as a sink in the global carbon cycle due to its recalcitrance [9,37]. Experiments on laboratory [38] and field scale [39–42] as well as investigations on soils containing pyrogenic carbon [4] show in many cases an improvement of soil characteristics, but also opposite results for a considerable number of others [43]. Interactions with soil and soil biota are very complex and strongly influenced by the physical and chemical properties of biochars [43,44]. The knowledge of these properties is important not only for the application of biochars as a soil amendment, but also in any technical application. This is not an easy task, because of the large diversity of production processes and the heterogeneous nature of feedstock and char [45,46]. Therefore, the testing, development and improvement of analytical methods is still a problem to be dealt with. Methods that allow fast and robust analysis for quality control are an inevitable requirement for a broad application of biochars. The choice of such methods must be supported by the combined use of different analytical methods including sophisticated and more laborious methods.

Although some general rules exist for comparing different production methods from different feedstock, the variance of the resulting biochars is still far from being understood. Charring temperature, heating rate and feedstock selection are key factors for the properties of biochars [47]. It was found that during the process of carbonization, the type of char changes over several steps from nearly unaltered feedstock to turbostratic graphitic carbon with increasing temperature [48]. Transitions between different types of char and the composition of chars depend on the feedstock. Biochars from grasses usually exhibit a larger amount of inorganic components [48]. Chars with large inorganic content require a more complex analytical strategy [49]. Analytical methods frequently used for the characterization of biochars are, among others [50], proximate and ultimate analysis [51], scanning electron microscopy (SEM) [52], X-ray diffraction (XRD) [53,54], X-ray photoelectron spectroscopy (XPS) [54,55], Raman spectroscopy [54], Fourier transform infrared spectroscopy (FT-IR) [56] and titration methods [57–59]. They yield information about the chemical composition, the structure and the surface functional groups of biochars. Reliable results on the relevant electrical conductivity of chars, which plays an important role for their electrochemical behavior [60], cannot easily be determined due to the porous nature of the material and the fact that the conductivity of carbon powders depends on the quality of the contact between the particles and on the pressure applied to the samples [61,62]. Since the electrical conductivity influences the polarizability of electron conductors, impedance or dielectric measurements can be in principle used for the determination of the conductivity and the degree of graphitization of carbon particles [15]. The low frequency impedance spectroscopy of rocks and soil is called spectral induced polarization (SIP) in geophysics. This method can be used to determine the polarization of electronically conductive particles that are disseminated in a matrix of other granular material and in contact with an electrolyte [63]. It has been proposed for the non-invasive characterization of biochar in soil [64–66].

In this study, two activated carbons and seven biochars from different feedstock and production processes were investigated. The objectives of this study were (i) evaluating the suitability of various analytical methods that are frequently used for studies on biochars, and (ii) characterizing the investigated chars by integrating the results of different methods. While pyrolysis and gasification are favorably used for the carbonization of dry matter like soft and hardwood, hydrothermal carbonization is predestined for wet materials like grasses. Regarding these preferences, biochars from soft and hardwood made by pyrolysis and gasification and from corn silage and miscanthus made by hydrothermal carbonization were investigated with respect to their chemical properties, structure, morphology and electrical polarizability. The terminology in this paper defines all carbonaceous materials used for this study as chars (including activated carbon). All chars excluding activated carbon are termed biochars. They are divided into hydrochars made by hydrothermal carbonization and pyrochars made by pyrolysis or gasification. The paper focusses on the characterization of oxygen containing surface functional groups and the state of carbonization and graphitization in connection with the electrical polarizability of biochar. It integrates results of several analytical methods, namely elemental analysis, pH and conductivity measurements, XPS, FT-IR, XRD, Raman spectroscopy, UV-Vis-spectrometry, scanning electron microscopy (SEM), and SIP and elucidates several drawbacks for the comparison of analytical methods for chars from different origin.

## Materials and methods

### Materials

#### Chars

The biochars used in this study were made from different feedstock using different processes. Four different samples were taken from the stock material of each of the seven biochars for the investigations in Belgrade (SEM, XRD, FT-IR, Raman, titrations), the chemical analyses, XPS, and SIP in Jülich. Subsamples were taken from these samples for each of the determinations. For each of the chemical analysis (C, H, N; oxygen; other elements), except ash determination, three subsamples were independently processed. All other measurements were usually made on single samples. Some measurements that yielded unexpected results (e.g. FT-IR on HW1100g, Raman on PW700g) were repeated, but were found to be reproducible. Two activated carbons were used as reference chars, AC1 (Fluka, particle size 75% ≤ 40 μm) and AC2 (Sigma Aldrich, particle size 36–150 μm). For the measurements with nitrogen adsorption and XPS, AC2 was washed several times with demineralized water in order to avoid the contamination of the instruments due to the potential sublimation of phosphorous pentoxide in the vacuum. SW500f was made via fast pyrolysis by Pytec GmbH, Hamburg, Germany, in an ablative reactor at 500 °C with a contact time of < 2 s from chopped spruce (*Picea spec*.) wood chips after drying to a moisture content of less than 10% [67]. HW500f was produced by fast pyrolysis of hard wood in a fluidized bed reactor at 500 °C with a contact time of < 2 s. It was received as a fine powder from Dynamotive USA, McLean, VA, USA [68]. BW550s was a commercial charcoal sold by proFagus GmbH, Bodenfelde, Germany. It was made from beech (*Fagus spec*.) wood pieces (< 30 cm) that where dried to a water content of 15–18% and pyrolized at 550 °C by use of the Degussa process in a retort with a pyrolysis time of typically 16–20 hours [67,69]. The large pieces were crushed and sieved. The size fraction between 0.125 and 2 mm was used for this study. HW1100g was made by gasification of chopped hard wood (90% beech and 10% oak (*Quercus spec*.)) after drying to less than 15% moisture in a downdraft gasifier at 1100 °C (in the oxidation zone). It was a fine powder obtained from Mothermik GmbH, Pfalzfeld, Germany [67]. PW700g was obtained from Carbon Terra GmbH, Augsburg, Germany. It was made from pine (*Pinus spec*.) wood chips in a Schottdorf kiln that operates with a temperature gradient from top (input of the feed material) to bottom (output of the biochar). Air was inserted from the bottom leading to gasification at 700 °C in the glowing zone near the output. The size fraction < 2mm was used for this study. CS180h was a mixed sample of corn (*Zea mays* L.) silage made from products of Artec Biotechnologie GmbH, Bad Königshofen, Germany and OWL University of Applied Sciences and Arts, Höxter, Germany. All components were produced by hydrothermal carbonization at 180 °C for typically 8–12 h. M200h was produced by hydrothermal carbonization of *Miscanthus x giganteus* biomass at 200 °C by Schlitt GmbH (Antrifttal-Ohmes, Germany) using HTC at 200 °C for typically 4–8 h.

#### Other materials

Sand F36 with 97% of the material having a grain size between 125–250 μm was obtained from Quarzwerke Hürth. All chemicals including NaCl, KBr, Na_2_HPO_4_·12H_2_O, NaOH, HCl used in this work were p.a. grade from Merck, Darmstadt, Germany.

### Methods

#### Scanning electron microscopy

Investigations on the morphology of the chars were carried out with a Jeol JSM-840A (Jeol, Akishima, Tokio, Japan) scanning electron microscope. The samples were fixed on an object slide with Carbon Conductive Paint from SPI Supplies, West Chester, PA, USA and sputter-coated with gold in a low vacuum atmosphere using a Fine Coat Ion Sputter JFC-1100 unit (Jeol, Akishima, Tokio, Japan).

#### Nitrogen adsorption

The specific surface area of the chars was determined with nitrogen adsorption by the Brunauer-Emmett-Teller method (BET) with an AUTOSORB-1 (Quantachrome Instruments, Boynton Beach, USA). Samples for BET were degassed in vacuum at 100 °C for 2 hours prior to the measurement to remove volatile compounds that could contaminate the apparatus. AC2 was first eluted with water to remove phosphoric acid and then pretreated as described above.

#### Chemical analysis

Element analyses for C, N and H were performed on a Vario EL cube (Elementar Analysensysteme GmbH, Langenselbold, Germany). All chars were dried at 106 °C until constant weight. Oxygen was determined with a LECO TCH600 (LECO, St. Joseph, MI, USA). The ash content was determined in a muffle furnace at 815 ± 15 °C. Three samples of each char were also analysed for 23 other elements by Inductive Coupled Plasma Optical Emission Spectroscopy (ICP-OES). To do so, 50 mg of sample were mixed with 0.25 g of LiBO_3_ and heated within 3 h to 1000 °C in a muffle furnace. The decomposition was performed for 30 min at this temperature. After cooling, the melt was dissolved in 30 ml HCl (5%). The solution was diluted to 50 ml. Duplicate samples were diluted 1/10 and analysed with an iCAP 7600 (Thermo Fisher Scientific, Waltham, Massachusetts, USA).

#### Ion chromatography

Soluble phosphate in AC2 was determined with ion chromatography. 0.2 g of AC2 was given to 10 ml Millipore water. The mixture was stirred with a magnetic stirrer at 900 rpm for 2 h. After centrifugation at 14000 rpm for 20 minutes, the supernatant was filtered using a nitrocellulose filter with pore size < 0.22 μm into a vial and kept in a freezer until analysis. The ion determination was performed on a Dionex ICS-3000 ion chromatographic set-up consisting of a single pump, a conductivity detector (ASRS ULTRAII (4 mm), recycle mode), an eluent generator with a Chromeleon® Chromatography Workstation and Chromeleon 6.7 Chromatography Management Software. All separations were performed at 30° C using a Dionex^™^ IonPac^™^ AG15 (4 x 50 mm, P/N 053942) and a Dionex^™^ IonPac^™^ AS15 IC (4 x 250 mm, P/N 053940) as guard and analytical columns. The mobile phase was 38 mM KOH with a continuous flow rate of 1 ml min^-1^. In addition, the column temperature was 30 °C, the conductivity cell temperature 35 °C and the suppressor current 95 mA. A backpressure of ≈18 MPa was applied. The calibration was performed with standard solutions of Na_2_HPO_4_·12H_2_O. The recovery rate was determined by spiking the sample with standard solutions of phosphate that were added to 0.2 g of AC2. The average of three recovery values was 97.1%. The linearity of the method was assessed using ordinary least-square regression.

#### X-ray diffraction

The X-ray powder diffraction investigation was conducted on a Rigaku Smartlab X-ray Diffractometer in θ-θ geometry (the sample in horizontal position) in parafocusing Bragg-Brentano geometry using D/teX Ultra 250 strip detector in 1D standard mode with CuK_α1,2_ (λ = 1.54178 Å) radiation source (U = 40 kV and I = 30 mA) and Ni-filtering. The XRD patterns were collected from 5–70° for 2θ, with a step width of 0.01°. Data collection speed was 2°/min with horizontal sample rotation of 20 rpm. A low background single crystal silicon sample holder was used to minimize the background. The crystalline materials present in the samples were identified in dedicated Rigaku PDXL 2.0 software, comparing them with ICDD PDF2-2016 standards.

#### EC and pH measurements

EC and pH were determined from leachates of 1 g of biochar in 10 ml demineralized water. The samples were shaken for 2 h at room temperature in a SM30 shaker (Edmund Bühler GmbH, Bodelshausen, Germany). Chars were separated with a Whatman Grade 40 ashless filter. EC and pH were measured with a Jenway 4330 (Jenway, Gransmore Green, Essex, England) with automatic temperature compensation for 25 °C.

#### Removal of acid soluble ash components

The chars were shaken for 24 h with 0.05 M HCl in order to remove ash components [57]. Then they were washed twice with 1 M CaCl_2_ and subsequently with deionized water (all at a 50/1 solution/char ratio) until the filtrates had an electrical conductivity < 10 μS /cm. Finally, the samples were dried for > 60 h at 50 °C and stored in weighing bottles in a desiccator over silica.

#### UV-Absorbance of basic leachates

A sample of 0.500 g of HCl-treated char was shaken with 25 ml of 0.05 mol l^-1^ NaOH for 24 h to extract organic components of low molecular weight. The solid was separated from the solution by filtration using 0.45 μm nitrocellulose filters. The absorbance at 250 nm (A250) was measured as a qualitative index of dissolved aromatic organic compounds in the filtered extracts after separation and addition of HCl to achieve a pH < 2 [57]. If needed, samples were diluted with deionized water to show absorbance below 1. The pH was adjusted in diluted solutions to values < 2. Spectra were recorded on a double-beam UV-Vis spectrometer Cintra 10E (GBC Scientific Equipment, Braeside, Victoria, Australia). Absorbance of diluted samples was recalculated with respect to the dilution factor.

#### X-ray photoelectron spectroscopy

All chars were ground with an IKA M20 impact mill for 5 min to achieve a suitable consistent particle size for this determination. AC2 was washed phosphate-free with water prior to grinding. HW110g was a fine powder which could also be investigated without grinding. XPS was measured on a Phi5000 VersaProbe II from ULVAC-Phi Inc., USA with a monochromatic Al K_α_ source at 1.486 keV, 50 W, 15 kV and with a 200 μm spot. For the survey spectra, a pass energy of 187.5 eV was set with a step width of 0.8 eV and 100 ms/step. For the detailed spectra, the pass energy was 23.5 eV, the step width 0.1 eV with 100 ms/step. A Shirley background was used for baseline correction and the amounts of elements or functional groups in atomic % (at%) were determined with an error of ≥ 10% as the fractions of signals normalized to the total signal area using empirical relative sensitivity factors. A charge correction was performed by setting the signal of the main C-C component of the C1s signal to 285 eV for sp^3^ or 284.5 eV for sp^2^. The indium peak found in the spectra was due to indium foil that was used to fix the powder sample on the sample holder. All samples were outgassed in vacuum before the XPS measurement 1 h at 100 °C and subsequently 2 h at 200 °C. All components used for the deconvolution of the peaks were symmetric signals except C1s C-C sp^2^. Reference data were taken from the literature [70].

#### FT-IR spectroscopy

Fourier transform infrared Spectroscopy (FT-IR) was used to identify structural chemical units of chars. The transmission of IR radiation was measured on diluted mixtures of chars in KBr pellets between 400 and 4000 cm^-1^ with a resolution of 1 cm^-1^. KBr was molten in a platinum crucible and immediately stored in a desiccator over silica, in order to avoid the uptake of moisture from air. All treated and untreated chars were dried at 50 °C for > 60 h and stored over silica in a desiccator. An amount of 150 mg of KBr were mixed with 1–2 mg of char. The mixture was powdered in an agate mortar and was pressed under vacuum in order to obtain a translucent pellet. Spectra were recorded on a Nicolet 6700 FT-IR (Thermo Fisher Scientific, Waltham, Massachusetts USA) with 1 cm^-1^ resolution. Data were evaluated with OMNIC software, Version 7.0. Spectra were expanded to values between 100 and 10% for the determined minima and maxima and therefore do not yield absolute values.

#### Raman spectroscopy

The micro-Raman spectra were taken in backscattering configuration and analyzed by a TriVista 557 spectrometer, equipped with a nitrogen-cooled charged-coupled-device detector in additive mode. A Verdi G optically pumped semiconductor laser from Coherent Inc., Santa Clara, CA with a wavelength of 532 nm and a power of 25 mW was used as the excitation source. Spectra were fitted with OriginPro 2019 (OriginLab Corporation, Northampton, MA, USA) after appropriate baseline correction as described in the Results.

#### Spectral induced polarization

SIP measurements were made using the equipment and design considerations presented by Zimmermann et al. [71]. They were performed in an air-conditioned room at 20.5 ± 1 °C. Conductivity values were corrected to 20 °C by assuming an increase of 2% per °C using the actual measured room temperature. A rectangular box with the top open to air was used as sample holder. The box was 60.5 cm long, 5.725 cm wide and 8 cm high. The distance of the current electrodes was 35.7 cm and the potential electrodes were placed symmetrically between them with a separation distance of 10.6 cm. Pieces of commercial bath sponges of 4 cm thickness were placed between the current electrodes and the sand-charcoal mixture in order to save the electrodes from soiling and to reduce the required amount of material. Ag/AgCl electrodes filled with 4 mM NaCl were used for potential measurement.

2% (w/w with respect to the mass of sand) of charcoal was added to air-dry sand and the materials were mixed by stirring with a spatula. The mixture was wetted with electrolyte solution and stirred again. The materials were filled into the sample holders in portions. Therefor, some electrolyte was first poured into the sample holders and then the solid materials were added so that a slight excess of liquid remained. This mixture was stirred with a spatula in order to remove air and ensure complete saturation. The rectangular sample holder was not completely filled. The space outside the bronze electrodes was only filled with electrolyte and the filling height of the solid material was about 2.5 cm. The exact height was measured with a caliper rule. Since the surface of the solid mixture was not exactly flat in the rectangular sample holder, the accuracy of the cell constant for calculating the conductivity had an error of about 10%.

## Results and discussion

### Scanning electron microscopy (SEM) and specific surface area

**Fig 1:**
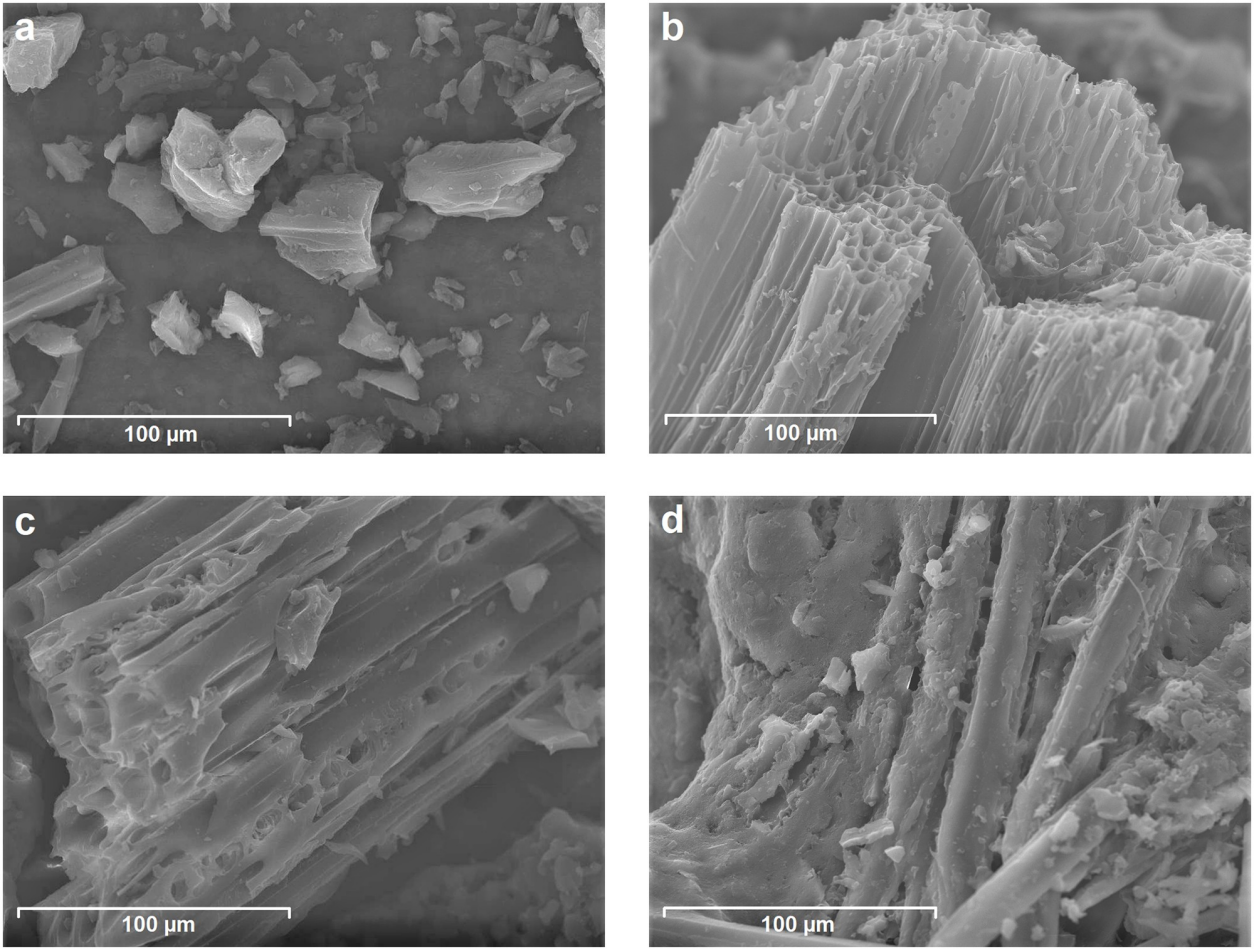
SEM images of selected chars: (a) AC2, (b) PW700g, (c), SW500f and (d) M200h.

SEM and BET were used to determine structural properties of the chars. Selected SEM images representing the main structural features found for different types of chars are presented in Fig 1.

Both activated carbons (AC1, AC2) consist of irregularly shaped particles, which exhibit layered structures with typical terraces. The size of the larger particles of AC2 (Fig 1a) corresponds to the size given by the provider (36–150 μm), but smaller particles are also visible. S1a Fig in S1 File shows the image of AC1 with a larger amount of smaller particles in accordance with the particle size (75% < 40 μm). Fig 1b shows the highly porous structure of PW700g which is typical for pyrolytic chars from slow pyrolysis or gasification. A similar structure is also observed for BW550s (S1b Fig in S1 File) and HW1100g (S1c Fig in S1 File), but with thicker pore walls which might be a consequence of the morphological properties of the actual piece or the different type of the feedstock (hardwood). In contrast, the porous structure of HW500f (Fig 1c) and SW500f (S1d Fig in S1 File) is less developed, because these biochars are not yet fully pyrolyzed. The fast pyrolysis biochars contain material of different structure, visible for SW500f in particular (S1d Fig in S1 File). The tubular pores exhibit less sharp edges, seem to be partially filled and exhibit hazy entrances (Fig 1c). Fig 1d illustrates the surface morphology of M200h. Similar to CS180h, (S1e Fig in S1 File) this hydrochar shows a fibrous structure. Precipitated smaller particles can be observed at the surface of the fibers. These particles were formed upon aggregation from sphere-like particles formed by the degradation of saccharides and cellulose components during hydrothermal carbonization [52,72,73].

The data of the specific surface area A_sp_ are presented in Table 1. A_sp_ was largest for activated carbons which are optimized for that property. Physical activation by gases like CO_2_ and chemical activation e.g. by phosphoric acid usually performed between 500 and 800 °C removes reactive material from carbonized precursors (biochars or lignite) and strongly enhances the amount of micropores forming a valuable adsorbent, catalyst or catalyst support with extremely high surface area. Relatively high surface areas were also found for biochars from gasification and BW550s which were intensively carbonized at higher temperatures and/or with longer treatment time. During carbonization they were in contact with activating gases like CO_2_ and steam, but under less controlled conditions. The hydrothermal products show much lower surface area due to the much lower reaction temperature but still higher values than biochars made by fast pyrolysis. The latter are known to contain large amount of tar which can block the entrance to small pores [28,74].

**Table 1:** Specific surface area determined by BET.

Char	A_sp_(BET) / m^2^ g^-1^
AC1	880
AC2	1200
SW500f^#^	< 0.4
HW500f	0.9
BW550s	39
HW1100g^#^	104
PW700g	170
CS180h	6.9
M200h	5.4

^#^ taken from Borchard et al. 2012 [67].

### Chemical analysis

Data for the mass fraction X of the main elements related to the mass of the incinerated char are given in Table 2. The concentrations of other elements are listed in Table 3. The sum parameters X_CHNOS_ (sum of the main elements), X_el_ (sum of other elements), X_all_ (sum of all analyzed elements), Y_ox_ (calculated value for the oxides corresponding to X_el_) and Y_ash_ (amount of ash determined in a single experiment) related to the mass of the analyzed char are shown in Table 4. The chemical composition of the chars differs depending on the production process and the feedstock. The analytical values are given as the mean value and the standard deviation of three determinations. The analytical relative errors of the determinations of single main elements and of the sums of C, H, N, O, and S calculated from those values were in most cases lower than 1% with the largest error for the oxygen content and the sum of the main elements of PW700g with about 9% for X_O_ and 2% for X_CHNOS_. The sums for all determined elements X_all_ (Table 4) were all near 100%, but the deviations for several chars cannot be explained by pure analytical errors. However, they may be owing to lacking elements that were not determined (for values below 100%) and the heterogeneity of the chars [45], because the determination of C, H and N as well as the determination of O and S, and the analysis of other elements were made from three different samples of the same chars. The largest deviation was found for HW500f, which has also high ash content, with 104.9 ± 1.4% for the sum of all determined elements.

**Table 2:** Composition of chars in weight per weight %.

Chars	X_C_/ %	X_H_/ %	X_N_/ %	X_O_/ %	X_S_/ %
AC1	85.4±0.38	0.96±0.03	0.63±0.05	6.9±0.1	0.719±0.01
AC2	77.1±0.53	3.51±0.04	< 0.4	17.8±0.8	< 0.01
SW500f	75.9±0.69	3.79±0.06	< 0.4	19.9±0.3	< 0.011
HW500f	70.0±0.06	3.53±0.09	< 0.4	25.2±1.3	< 0.012
BW550s	86.8±0.37	2.85±0.03	< 0.4	8.47±0.03	< 0.013
HW1100g	80.6±0.58	1.70±0.03	< 0.4	11.5±0.7	< 0.013
PW700g	62.1±0.28	1.71±0.05	0.91±0.06	23±2	0.034±0.005
CS180h	58.6±0.14	5.98±0.12	1.86±0.05	33.5±0.2	< 0.013
M200h	50.5±0.27	5.78±0.05	< 0.4	39.6±0.4	< 0.013

**Table 3:** Elements determined by ICP-OES.

Chars	Al/ mg·kg^-1^	Ba/ mg·kg^-1^	Ca/ mg·kg^-1^	Cr/ mg·kg^-1^	Fe/ mg·kg^-1^	K/ mg·kg^-1^	Mg/ mg·kg^-1^	Mn/ mg·kg^-1^	Na/ mg·kg^-1^	P/ mg·kg^-1^	Si/ mg·kg^-1^	Sr/ mg·kg^-1^	Ti/ mg·kg^-1^	Zn/ mg·kg^-1^	Zr/ mg·kg^-1^
AC1	9100 ±300	62.4 ±1.4	188 ±10	< 20	1250 ±40	870 ±30	269 ±8	5.8 ±0.4	241 ±16	< 70	13900 ±400	< 20	421 ±16	< 2	26 ±2
AC2	< 10	< 3	107 ±9	< 20	39 ±13	< 10	17.9 ±0.7	5.4 ±0.5	< 20	15880 ±190	139 ±8	< 20	< 5	4.3 + 0.6	28 ±2
SW500f	< 10	64.5 ±0.9	2846 ±8	153 ±6	2844 ±14	2297 ±19	747 ±2	528.4 ±1.4	1125 ±14	490 ±40	881 ±9	< 20	< 5	87 ±1.6	4.8 ±0.1
HW500f	620 ±16	69.6 ±1.2	5170 ±90	262 ±8	1020 ±18	5900 ±80	726 ±11	179 ±3	< 20	270 ±30	47300 ±700	< 20	673 ±19	14 ±2	33.5 ±1.8
BW550s	918 ±14	61.53 ±14	3020 ±30	< 20	95 ±13	2960 ±20	737 ±3	360.7 ±1.4	< 20	270 ±30	247.5 ±9.6	< 20	< 5	7.1 ±1.3	9.9 ±0.9
HW1100g	975 ±15	79.7 ±0.9	25200 ±300	< 20	877 ±12	12450 ±50	3872 ±17	769.5 ±1.9	674 ±13	1200 ±30	3510 ±20	112 ±16	61 ±10	109.8 ±1.9	13.5 ±1.1
PW700g	5880 ±120	292.3 ±0.8	33400 ±500	1133 ±8	6097 ±13	10610 ±40	4032 ±16	3928 ±7	1430 ±13	2764 ±15	53500 ±400	136 ±16	745 ±11	293.1 ±9.9	35.8 ±0.8
CS180h	< 9	< 3	950 ±40	< 20	4280 ±140	4090 ±120	670 ±20	24.06 ±0.97	< 20	1210 ±30	3420 ±120	< 20	< 5	14 ±2	11.6 ±1.7
M200h	< 10	< 3	7030 ±100	< 20	1459 ±18	7126 ±96	3860 ±50	59.48 ±0.99	< 20	510 ±20	6330 ±90	< 20	< 5	16.4 ±1.7	< 5

**Table 4:** Sum parameters of the chemical analysis.

Chars	X_CHNOS_ / %	X_el_ / %	X_all_ / %	Y_ox_ / %	Y_ash_ / %
AC1	94.58±0.40	2.633±0.050	97.2±0.4	5.161	6.53
AC2	98.41±1.04	1.622±0.019	100.0±1.0	3.697	0.65
SW500f	99.59±0.85	1.207±0.016	100.8±0.8	1.782	2.06
HW500f	98.73±1.36	6.224±0.071	104.9±1.4	12.191	18.1
BW550s	98.12±0.55	0.869±0.006	99.0±0.6	1.269	1.69
HW1100g	93.80±0.99	4.990±0.031	98.8±1.0	7.264	8.83
PW700g	87.75±2.02	12.428±0.065	100.2±2.0	21.875	19.4
CS180h	99.92±0.27	1.467±0.023	101.4±0.3	2.365	4.04
M200h	95.88±0.63	2.639±0.018	98.5±0.6	4.173	5.27

**Fig 2:**
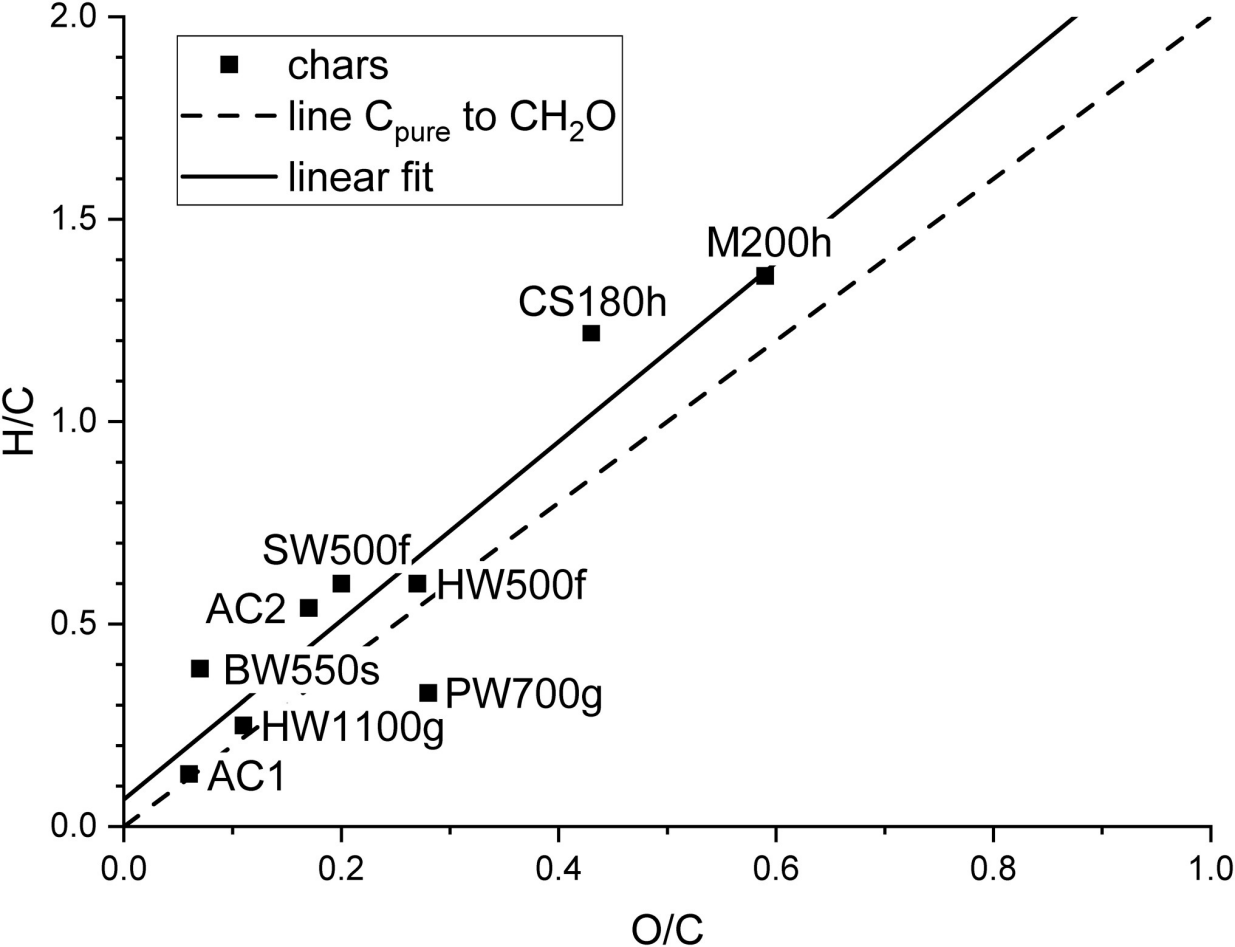
Van-Krevelen diagram of the investigated chars.

The nitrogen content was below the detection limit for most of the chars and generally more than one order of magnitude lower than the oxygen content. Therefore, it does not play an important role for the properties of the chars considered in this paper. Sulphur was above the detection limit for AC1 and PW700g and will be discussed later. The content of C, H and O is best discussed by a van Krevelen diagram (Fig 2) [52,75]. All chars except PW700g show a higher H/C and a lower O/C ratio than expected for the loss of water from formal carbohydrate (CH_2_O), the elementary building block of plant biomass, as represented by the dashed line in Fig 2. Whereas the hydrochars exhibit large H/C and O/C ratios, the activated carbons and the pyrochars show considerably lower values indicating a larger degree of dehydration. The H/C ratio decreases with increasing temperature for the pyrogenic chars. The relatively low value for BW550s is due to the much larger contact time. The trend in the van Krevelen diagram corresponds quite well with that shown by Weber and Quicker [76] for slow pyrolysis irrespective of the different production processes for the chars used in this study. Despite the different initial O/C and H/C ratios of the feedstock, the linear fit of the data has a slope of 2.2, which is close to the value of 2 that is expected for mere dehydration which is obviously the main process during the thermal decomposition of plant biomass. The intercept is 0.067 and represents the mean influence of the initial composition of the biomass and the changes due thermal degradation. Decarboxylation and decarbonylation result in lower O/C and higher H/C, dehydrogenation in lower H/C and demethanization in lower H/C and higher O/C. Evolution of other volatile compounds also affect O/C and H/C. The largest deviation from the linear fit is found for CS180h and PW700g. Although CS180h was made at lower temperature, the degree of carbonization is higher than for M200h for which the composition is similar to values in the literature [77]. This might be a consequence of a longer reaction time. The high oxygen value of PW700g is partially due to the high ash content. The upper limit of the oxygen bound in the inorganic components can be calculated as an approximate value from the difference of X_el_ and Y_ox_ calculated from the corresponding oxides of X_el_ (Table 4). The corresponding O/C molar ratio excluding the maximum oxygen in the inorganic compounds would be 0.19, which is much closer to the dashed line in Fig 2. A similar shift would be found for ash-rich HW500f resulting in an O/C ratio similar to SW500f.

The largest relative standard deviations for the oxygen content were found for the biochars with the largest ash content HW500f (18.1%) and PW700g (19.4%). This might be an indication that the ash components are inhomogeneously distributed in the chars. Both ash-rich chars also contain considerable amounts of silicon as can be seen from the content of further elements in Table 3. The high silicon content of both chars is probably due to a considerable contamination with silicon-rich material. In the case of HW500f, it may be sand from the fluidized bed in the reactor. The contamination of PW700g is probably due to soil, because also other soil-typical elements (Al, Ca, Fe, K, Mn, Na) show enhanced concentrations. AC 1 also contains considerable amounts of silicon (1.4%) and in addition aluminium (0.91%), sulfur (0.72%) and iron (0.13%). This can be expected for chars made from lignite or peat. Silicon is also present in most of the other chars with more than 0.1%.

Most of the other additional elements of Table 3 are only present as traces in most of the chars, only Ca, Fe, K, are found in most of the chars with more than 0.1%. All biochars have potassium content and, with the exception of CS180h, calcium content larger than 0.2%, whereas the activated carbons have lower values. The biochars from gasification have potassium values above 1% and also contain more than 2.5% Ca. They also have elevated amounts of other elements due to the removal of C, H and O and the enrichment of non- or less volatile components at higher temperatures. A large content of phosphorous (1.6%) was found in AC2, although the content of ash in AC2 is very low, indicating that phosphorus disappeared during the combustion for the ash determination. Therefore, it cannot have been present as phosphate, but as phosphoric acid which decomposes above 213 °C by loss of water. The resulting P_4_O_10_ sublimes above 362 °C and is not part of the ash. Consequently, Y_ash_ is lower than Y_ox_ for AC2 in contrast to all other chars except PW700g, which seems to be most affected by the inhomogeneous distribution of ash components due to its high ash content. The higher values for Y_ash_ than Y_ox_ are reasonable because some ash forming elements might not be present as oxides, but as carbonates or sulfates, whereas phosphates would be counted correctly in Y_ox_. Phosphorous content above 0.1% is also found in both gasification chars and in CS180h.

In order to confirm the presence of soluble phosphorous in the original activated carbon AC2, the amount of phosphate from an eluate was determined with ion chromatography. The lower limit for the content of soluble phosphate obtained from a single extraction of AC2 was 19 g kg^-1^, which is about 50% of the total P found by chemical analysis.

Overall, the determination of the main and the inorganic elements of the chars yielded reliable values. The results give an impression of the large variety of composition that can be found in chars of different origin and on the potential influence of extraneous material from the feedstock or the processes. The analysis of the inorganic components is inevitable at least for chars with high ash content and for chars containing volatile inorganic components like AC2.

### X-ray diffraction

**Fig 3:**
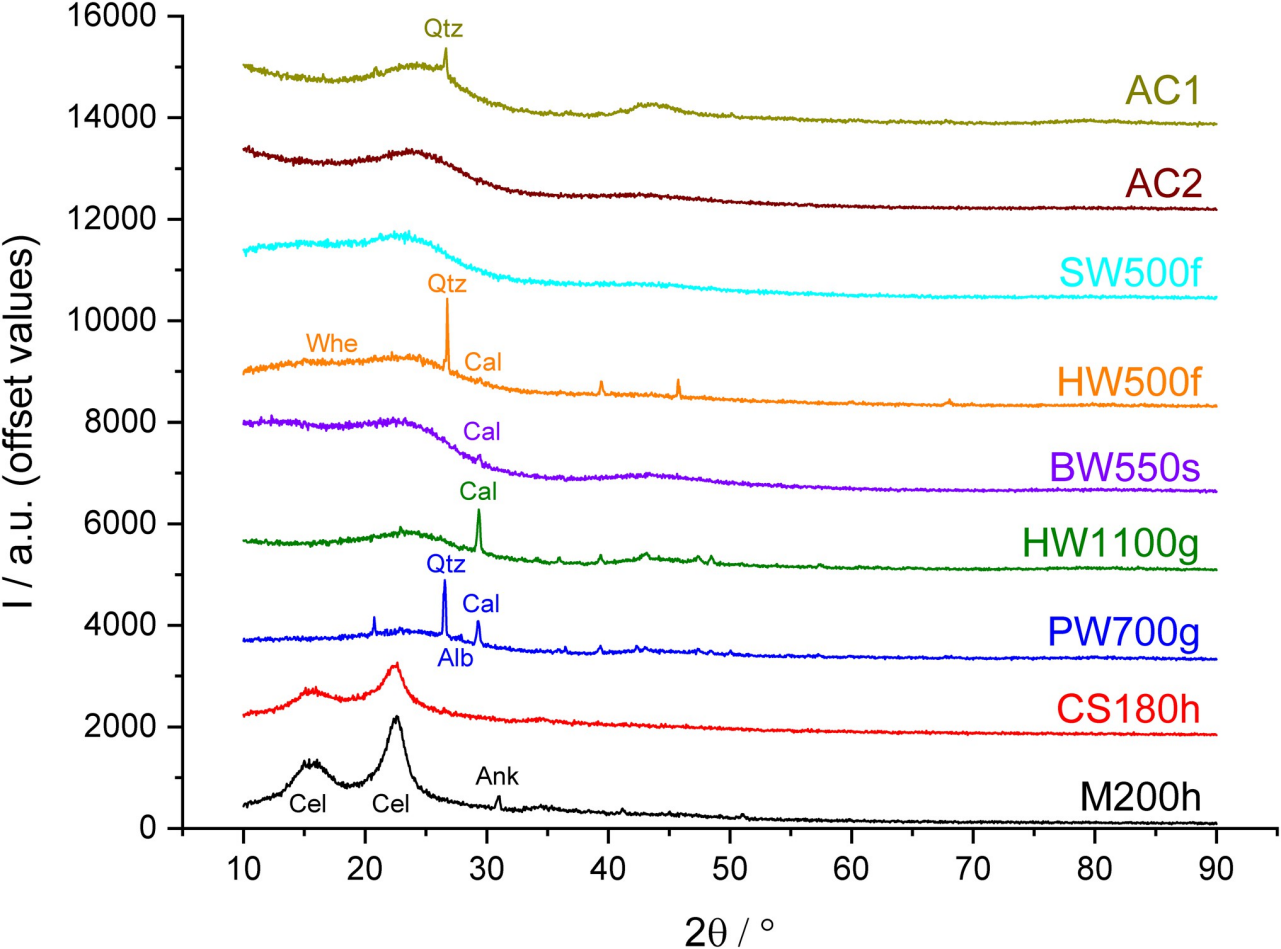
X-ray diffractograms of the chars.

X-ray diffractograms are shown in Fig 3 and with higher resolution in S2 Fig in S1 File. The scattering angles are given as values of 2θ and for better comparison with some literature also as distances in Å in the text.

The hydrochar samples CS180h and M200h show broad peaks at approximately 16.0°, 22.6° and 34° typical for cellulose and the corresponding hydrochar [54]. The sharp peak at 30.95° (2.889 Å) for M200h, can be attributed to ankerite (Ca(Fe^2+^,Mg,Mn)(CO_3_)_2_) [53] which is in agreement with the chemical composition of the sample (Table 3). CS180h does not show the peak for ankerite, in agreement with its much lower Ca-content (Table 3), or any other sharp peak.

XRD-patterns of the activated carbons and pyrochars show broad peaks of turbostratic carbon and sharp peaks of minerals of varying intensity, with the exception of AC2 and SW500f, which show no mineral peaks. Most of the mineral peaks belong to quartz and calcite with the most prominent peaks at 26.62° (3.348 Å) and 29.36° (3,042 Å) respectively [53].

The activated carbons are characterized by a strong decrease of intensity at low angles, which is typical for amorphous materials and two broad peaks at around 24° and 43.5° due to the presence of turbostratic carbon. AC1 also shows a sharp peak of quartz in agreement with the high Si content (Table 3). AC2 shows no sharp peaks and the broad peak at 43.5° is hardly visible similar to XRD patterns of some carbons activated with small amounts of phosphoric acid described in the literature [78].

XRD-patterns for fast pyrolysis biochars show a broad double peak for amorphous carbon between 5 and 29°. In addition, HW500f shows sharp intense peaks of quartz and small peaks for Whewellite (Ca(C_2_O_4_)∙H_2_O) at 24.38° and 14.86° (3.650 and 2.987 Å) [53] and calcite. The presence of Si and Ca is consistent with the chemical composition (Table 3). Other sharp peaks found in the spectrum could not be identified, but may result from other inorganic components in that biochar with high ash content (18.1%). X-ray diffraction of spruce wood-derived biochar SW500f with low ash content (2.06%) shows no sharp peaks, and the broad double peak is less pronounced compared to HW500f. The broad peak at lower angle may be a strongly disordered relict of cellulose in these chars [48], which were made with a short contact time at 500 °C.

The X-ray diffractogram of slow pyrolysis biochar BW550s also shows a broad double peak from 5–29°. The lower peak is shifted to lower angles compared with the fast pyrolysis biochars and seems to have another source, because relicts of cellulose can be excluded due to the long contact time at 550 °C. The reason for this peak could not be identified. For BW550s, a weak peak for calcite, but none for quartz is found in accordance with the chemical composition (Table 3).

The gasification biochars PW700g and HW1100g show a broad single peak between 15 and 29° and a variety of sharp peaks. Most of them can be attributed to quartz and calcite. The much lower intensity of the quartz peak for HW1100g relative to the calcite peak and with respect to PW700g is in accordance with the relative amount of Si and Ca in both biochars (Table 3). PW700g shows several other peaks from which only albite at 27.92° could be identified.

#### Leaching of inorganic and organic components

The electrical conductivity (EC) and the pH values of the aqueous leachates of the different chars are shown in Table 5 together with the UV absorbance of acidified basic leachates.

**Table 5:** Value of pH, electrical conductivity (EC) and UV absorbance determined at 250nm for the different chars.

Char	pH	EC / μS·cm^-1^	A_250_
AC1	6.1	69	0.10
AC2	2.1	3290	0.15
SW500f	4.1	360	2.09
HW500f	5.6	560	1.38
BW550s	6.3	180	0.05
HW1100g	9.9	1090	0.09
PW700g	7.9	740	0.70
CS180h	4.1	1080	10.5
M200h	6.0	1020	8.39

Whereas the leachates of chars from pyrolysis and HTC have pH values lower than 7, those of chars from gasification are alkaline. The leachate of AC1 is nearly neutral. In contrast, AC2 shows a strongly acidic reaction. The EC values are high for AC2, both hydrochars and HW1100g. The low pH and the high EC of AC2 is due to the activation of AC2 by phosphoric acid. Assuming that phosphate was present as phosphoric acid, the eluted amount of phosphate (19 g kg^-1^) corresponds to the pH of 2.1 found for AC2. Soluble compounds of ash-forming elements like K, Na, Ca, Mg and Fe increase EC and pH values. Both hydrochars exhibit high EC values. They are made from green feedstock which contains more sap and consequently more alkaline and earth alkaline ions than wood in the original biomass. Therefore, the ash-content of the hydrochars is comparable to that of pyrochars or even higher (Table 4) despite the fact that the ash content increases with increasing carbonization due to the loss of volatile elements. Inorganic ions from hydrochars should also more readily dissolve than those from pyrochars where they might already be fixed in insoluble minerals. The EC value for PW700g is intermediate, although the ash content is much higher than for HW1100g (Table 4). The main difference between both chars is the much higher silicon content in PW700g (Table 3). The difference in pH between HW1100g (9.9) and PW700g (7.9) is also in accordance with this finding. The pyrochars made at lower temperature release less ions corresponding to low ash content in SW500f as well as in BW550 and the dominant content of SiO_2_ in the ash of HW500f. The leachate of AC1 with high content of Al and Si has a particularly low EC value.

The UV absorbance of acidified NaOH extracts at 250 nm (A_250_) is presumed to correlate well with aromatic dissolved organic carbon [79]. Absorbance is high for the biochars made with fast pyrolysis and particularly high for both hydrochars. Such large A_250_ values (Table 5) are connected with large H/C atomic ratios for those biochars (Fig 2). According to Preston and Schmidt [80] this indicates the presence of less condensed structures and a higher proportion of thermally unstable organic substance of lower molecular weight. Differences between both pairs of char (PW500f/HW500f and C180h/M200h) may be the result of the different feedstock or differences in the details of the production process. PW700g also shows relatively high values of A_250_. Extracts of activated carbons show reduced absorbance and those of BW550s as well as HW1100g very low absorbance. BW550s was made by pyrolysis with a much longer contact time than all other chars and HW1100g was produced at the highest temperature. These results reflect the fact that both longer contact time and higher temperature lead to stronger carbonization. The low absorbance of BW550s demonstrates a low content of dissolvable organic carbon in this biochar, which also exhibits low ash content (1.69%). This suggests that most of the C in the feedstock was converted to fixed carbon during the long reaction time in accordance with the fact that this commercial charcoal was optimized for the use as a fuel.

### X-ray photoelectron spectroscopy

XPS yields results on the chemical composition in a thin layer at the surface of material, these results may be considerably different from those of the chemical analysis of bulk materials. Despite that fact, the overview of the elements (Table 6) at the surface of the chars qualitatively corresponds to the abundance found by chemical analysis and by XRD. The deviation of the O/C ratio from XPS and element analysis (Fig 2) was largest for the activated carbons and PW700g. The lower O/C ratio for AC2 obtained by XPS can be explained by the removal of phosphoric acid. HW1100g, which was obtained as a fine powder, was also investigated without grinding. The values for the non-treated sample HW1100g* and the ground sample HW1100g are nearly identical with respect to C1s and O1s, but considerably different concerning the fraction of different structural elements.

**Table 6:** XPS survey on different elements at the surface of ground chars and HW1100g* without pretreatment.

Char	C1s /%	N1s /%	O1s /%	Si2p /%	S2p /%	P2p /%	Ca2p /%	Ni2p /%	Mo3d /%
AC1	89.0		10.1	0.5	0.1			0.4	
AC2	90.5		9.2			0.3			
SW500f	78.9		20.9					0.1	0.1
HW500f	78.5^#^		20.6	0.6			0.3		
BW550s	92.4^#^		7.6						
HW1100g	89.6^#^		9.6				0.6	0.2	
HW1100g*	89.9^#^		9.6				0.5		
PW700g	86.3^#^		12.0	0.8			1.0		
CS180h	72.3	1.7	25.7	0.2					
M200h	72.0	1.0	26.4	0.3			0.3		

* not ground.

^#^ values include small amounts of K2p.

Large fractions of sp^2^ hybridized carbon were found from C1s data (Table 7) in AC1 and for the pyrochars made by gasification, whereas a relatively large amount of sp^3^ hybridized carbon was present in AC2 that was activated by phosphoric acid. The values of AC2 were similar to those of BW550s with ca. 50% sp^2^ and ca. 30% sp^3^. Very low values of sp^2^ were found for the hydrochars and low values for the pyrochars made by fast pyrolysis. For these two types of biochar large fractions of oxygen functional groups (C-O, C = O and COO) were found. The hydrochars also contain a large amount of sp^3^ hybridized carbon. A signal for π-π* satellites could not be detected for the hydrochars corresponding to the very low sp^2^ hybridization. This result shows that the carbonization of hydrochars was very low. The fraction of sp^2^ hybridized carbon and carbon of oxygen functional groups increased in the ground sample of HW1100g indicating that the creation of new surfaces influences the results of XPS. The result of C1s (Table 7) and O1s (Table 8) for C = O and C-O, however, are contradictory indicating that the chosen standard evaluation does not yield unambiguous results for the relative quantities of different oxygen functional groups. The values for C1s suggest a weak increase of C-O and a relatively strong increase of C = O, whereas the fraction of COO (carboxylic moieties) remains unaltered in the ground product. The fractions of O1s yield a strong increase for C-O and a decrease of C = O after grinding. The O1s signal of HW1100g* contains an additional signal (O1s?) that could not be identified. It may come from metal oxides that are enriched at the outside of the particles. This signal disappears after grinding probably due to a relative enrichment of material that is rich in carbon oxygen bonds at the freshly formed surfaces. Owing to the interference by the unidentified O1s signal, the results of the C1s signal seem to be more reliable for the discrimination of C = O and C-O moieties. The fraction of C-O is higher than the fraction of C = O for all chars except AC1. The fraction of COO is lowest for all chars except AC2. This result indicates that alcohols and phenols and their derivatives like ethers and esters are preferably present in the chars as surface functional groups in accordance with results on activated carbon in the literature [81]. The content of such groups is particularly high in the hydrochars and the biochars made by fast pyrolysis. For hydrochars, it is quite clear from XRD that there is still a large amount of unaltered cellulose present. For SW500f and HW500f there might also be an incomplete carbonization in accordance with the double peak found by XRD, but phenols on the carbon matrix may also have been formed in larger amount.

**Table 7:** XPS of C1s at the surface of ground chars and sample HW1100g* without pretreatment.

Char	C-C sp^2^ /%	C-C sp^3^ /%	C-O /%	C = O /%	COO /%	π-π* /%	K2p3 /%	K2p1 /%
AC1	86.1	0.0	2.5	3.0	2.4	5.9		
AC2	51.0	30.1	9.0	2.8	3.6	3.5		
SW500f	22.3	32.4	34.6	6.2	3.0	1.5		
HW500f	31.3	30.9	28.4	3.8	3.2	2.1	0.2	0.1
BW550s	55.1	31.0	5.2	2.7	2.1	3.7	0.1	0.1
HW1100g	77.9	9.5	2.8	2.4	1.3	5.3	0.5	0.3
HW1100g*	68.1	21.0	2.6	1.3	1.3	4.6	0.7	0.4
PW700g	75.9	11.0	3.5	2.6	1.6	5.2	0.2	0.1
CS180h	7.9	50.8	28.8	7.5	5.0			
M200h	8.7	46.6	33.9	6.4	4.4			

* not ground.

**Table 8:** XPS of O1s at the surface of ground chars and sample HW1100g* without pretreatment.

Char	C = O /%	C-O /%	H_2_O /%	O_2_/C /%	O1s? /%	R_O1s_(CO) /%	R_C1s_(CO) /%
AC1	27.2	41.6	22.6	8.6		1.53	1.41
AC2	34.8	57.3	5	2.8		1.65	1.49
SW500f	14.2	83.8	1.9			5.90	4.87
HW500f	23.4	76.6				3.27	2.88
BW550s	47.1	50.7	2.3			1.08	1.03
HW1100g	51.4	41.7	6.9			0.81	0.79
HW1100g*	56.3	19.1	1.7		22.9	0.34	0.33
PW700g	46.4	48.1	5.5			1.04	1.00
CS180h	19	81				4.26	3.38
M200h	12.5	87.5				7.00	5.18

* not ground.

Despite the degassing of the materials prior to measurement, a large amount of water was found in AC1 and still a considerable amount of water in all other samples except the hydrochars and HW500f (Table 8). The activated carbons also contained adsorbed oxygen (O_2_/C). With the exception of SW500f, adsorption of water and oxygen was preferably found for the chars with high specific surface areas. Whereas the influence of adsorbed water and oxygen on the sum of surface functional groups is minor for most of the chars, about 30% of the O1s fraction is due to H_2_O and O_2_ for AC1. Therefore, the corrected value for the total O1s fraction due to surface functional groups would be only about 0.07 instead of 0.1. The resulting O/C ratio is much closer to the value found by element analysis (0.06). The results for the relative fraction of different types of CO functional groups obtained by C1s and O1s are in fair agreement. The ratio of C-O and C = O from O1s, denoted as R_O1s_(CO) and the ratio of C-O groups and the sum of C = O and COO from C1s, denoted as R_C1s_(CO) in Table 8 are very similar for the gasification chars and BW550s, i.e. the highly carbonized non-activated chars. The difference is a bit larger for the activated carbons and further increases for the fast pyrolysis biochars. It is largest for the hydrochars and roughly increases with the fraction of carboxylic moieties, that probably overlap both signals of C-O and C = O of O1s.

Overall, XPS yields reliable results on the total fraction of oxygen functional groups at the surface of chars, but the results may be influenced by sample preparation and have a relatively large error. Unfortunately, a discrimination of alcohols and phenols and their derivatives (esters, ethers) is not possible by XPS. Since all those functional groups exhibit strongly different chemical behavior, the characterization of biochars by XPS alone is limited.

### Fourier-transform infrared spectroscopy

Since the examined biochars were obtained by different procedures and from different feedstock, they exhibit large differences concerning the intensities in different wavenumber ranges, and it is therefore impossible to present the FT-IR spectra in a single plot. For this reason, they are compared according to the type of pyrolysis. The quality of the spectra is considerably reduced in comparison with spectra of well-defined molecular compounds. The structural and reflective properties of the chars and the presence of a variety of overlaying bands lead to less resolved IR bands of relatively low intensity [82]. Artefacts in the determined spectra further reduce the quality of the spectra. All spectra contain positive or negative double peaks of CO_2_ in air at about 2300 cm^-1^. They are a consequence of the improper compensation of the background spectra due to varying CO_2_ content in air and/or a considerable intensity loss of IR radiation between sample and detector by the strong scattering effect of chars. The spectra of highly carbonized materials AC1, BW550s, HW1100g and PW700g show wiggles, in particular in the regions between 3300–4000 cm^-1^ and 1700–2000 cm^-1^. These artefacts may be due to the interference of IR radiation in the porous carbon materials or they may result from the electrical conductivity of the materials. The wiggles hamper the identification and interpretation of potential small peaks in those ranges from free phenolic OH, carboxylic compounds (acids, esters, lactones) and overtones of the aromatic hydrocarbons. Artefacts may also be observed between 2800–3000 cm^-1^ as presented in a typical background spectrum in the Nicolet FT-IR User’s Guide [83]. They are particularly critical because they overlay with bands from CH stretch vibrations of aliphatic groups.

Spectral features are discussed using potential structural units in accordance with the chemical and XPS analyses. They include the aromatic matrix, aliphatic and olefinic structures as well as oxygen functional groups [84] and inorganic components. The spectra were taken from untreated biochars and from biochars which were treated with HCl. For the activated carbons and the hydrochars, the spectra of treated and untreated samples were nearly identical, whereas for the pyrochars they were not. All spectra are dominated by absorption in two main wavenumber ranges. Absorption between 1450 and 1800 cm^-1^ can result from C = O stretching vibrations, skeletal vibrations of aromatic and heteroaromatic rings, C = C stretching vibrations of olefinic carbon and CH_3_ and CH_2_ asymmetric deformation vibrations [81,85]. Stretching vibrations from C-O in ethers, cyclic ethers, epoxides, phenols, and alcohols as well as OH and symmetric CH_3_ deformation vibrations are located in the range between 900 and 1450 cm^-1^ [81,85]. All chars also show considerable absorption and most of them a strong decrease of transmittance τ above 1700 cm^-1^ with minima above 3000 cm^-1^, which are due to the absorption of OH groups from alcohols, phenols and carboxylic acids. The peaks of the spectra are summarized in S1 Table in S1 File and shortly discussed in the Supporting Information. Those data however do not allow an adequate discussion due to the strong overlap of peaks in particular for strongly carbonized materials.

#### Activated carbons

**Fig 4:**
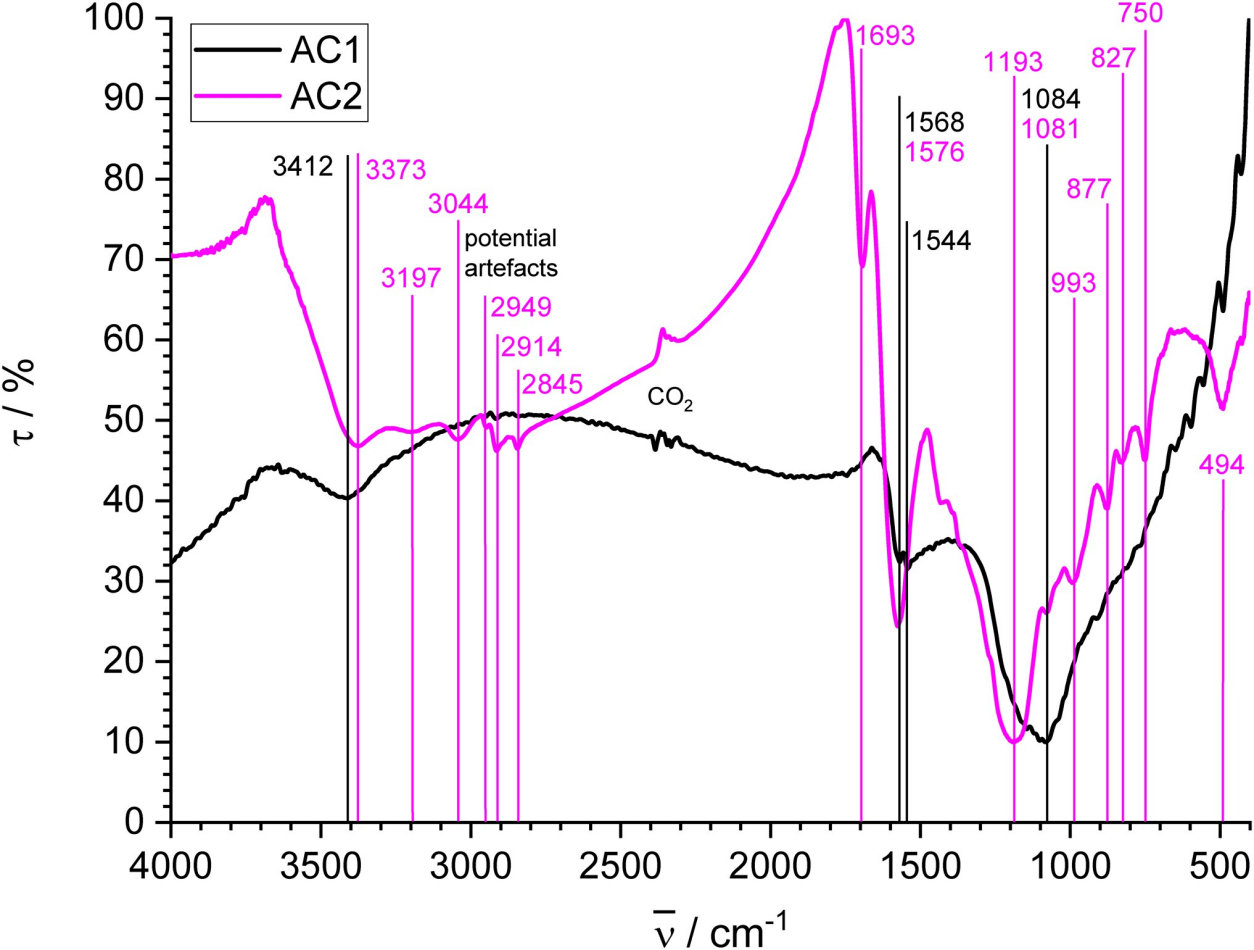
FT-IR spectra of activated carbons AC1 and AC2.

The FT-IR spectra of activated carbons AC1 and AC2 (Fig 4) look significantly different. Whereas several distinct bands for AC2 are well resolved, only broad unstructured bands are obtained for AC1, which has the lowest O/C and by far the lowest H/C ratio of all examined chars. The loss of intensity in highly graphitized materials is due to the reduced change of the dipole moments in the symmetric crystallites of graphitized carbon [86]. Broad bands of OH stretching vibrations with transmission minima at 3412 cm^-1^ for AC1 and 3373 cm^-1^ for AC2 occur in both cases [81]. AC2 exhibits a second flat minimum at 3197 cm^-1^ that might be due to another type of OH groups. The very broad range of low transmission around 3000 cm^-1^ for AC2 also suggests the presence of sorbed water [87,88], which may be associated with phosphoric groups on the surface. Additionally, AC2 has a band for aromatic CH stretching vibration at 3044 cm^-1^ [89]. In contrast, no distinct band for aromatic CH is found in AC1 in accordance with spectra of physically activated carbon in the literature, which also exhibit poor structure [86]. Bands at 2949, 2914 and 2845 cm^-1^ for AC2 could be attributed to aliphatic CH stretching vibrations, which have been found for carbons activated with phosphoric acid, in particular for those made at low temperature [89]. The peaks are not very strong and could also be influenced by the instrumental artefacts mentioned above, but they agree with the relatively large amount of sp^3^ hybridized carbon found by XPS.

The band for AC2 at 1693 cm^-1^ is usually attributed to the C = O stretching vibration in carboxylic structures [85,89]. A corresponding band is not present in the spectrum of AC1 despite a comparable amount of COO groups that is 2/3 of those found by XPS in AC2. This result suggests, that such peaks might be suppressed for chars with higher amount of sp^2^ carbon. The band at 1576 cm^-1^ for AC2 and the double band at 1568 and 1544 cm^-1^ for AC1 are associated with aromatic skeletal vibrations. Both activated carbons show broad bands of overlapping peaks with a minimum at 1193 cm^-1^ for AC2 and 1084 cm^-1^ for AC1. The band at 1081 cm^-1^ for AC2 is frequently assigned to C-O stretching, but may also result from symmetrical P-O vibration in P-O-P chain structures or P^+^-O^-^ in acid phosphate esters [89]. Bands at 993, 877, 827 and 750 cm^-1^ for AC2 are attributed to aromatic out-of-plain CH deformation [89] and correspond to the band at 3044 cm^-1^. In contrast, AC1 shows no distinct peaks of aromatic out-of-plain CH deformation vibrations between 700 and 900 cm^-1^ in accordance with the missing peak for aromatic CH stretching vibrations and the very low hydrogen content. The prominent band at 494 cm^-1^ for AC2 can be assigned to P-O deformation vibrations in phosphoric acid [90].

#### Fast pyrolysis biochars

**Fig 5:**
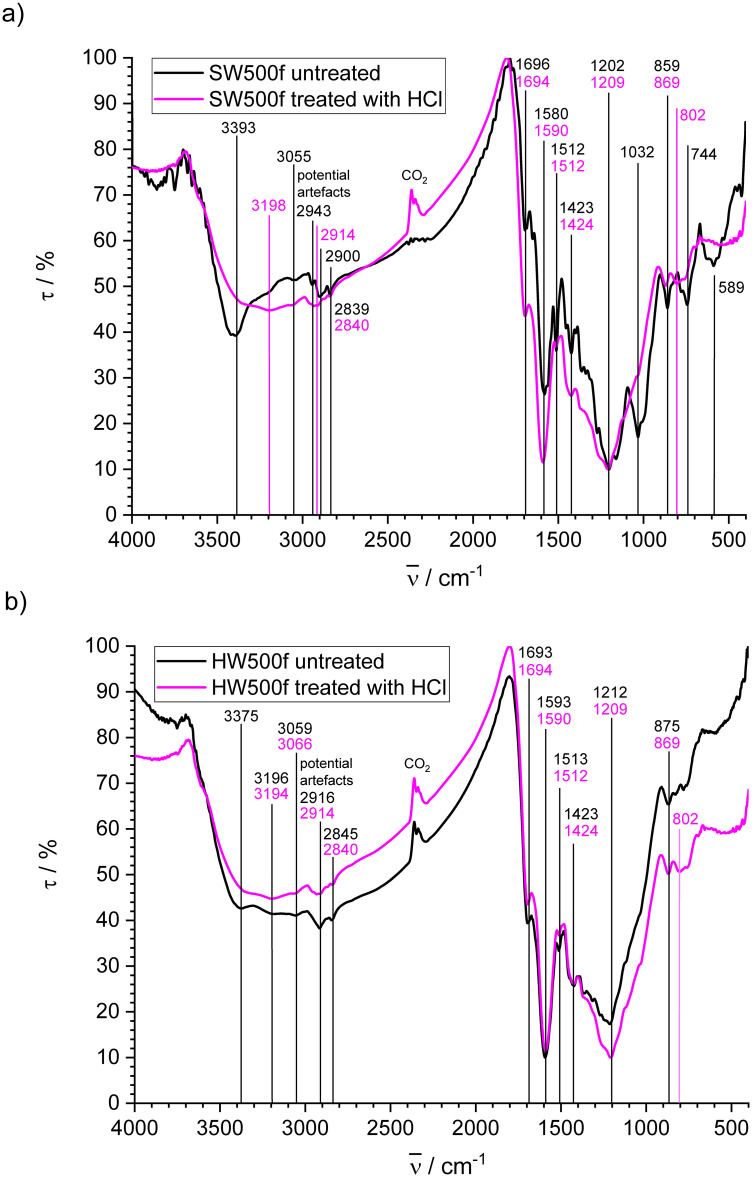
FT-IR spectra of biochars made by fast pyrolysis untreated and treated with HCl; a) SW500f; b) HW500f.

Fig 5a presents the FT-IR spectra of untreated and treated SW500f, Fig 5b those of HW500f. The spectra for these two biochars, which have high H/C and O/C ratio, are similar in shape and show some distinct peaks, which will be mentioned here preferably with the wavenumbers of the untreated samples, with few exceptions. Broad bands from OH stretching vibrations of phenolic and carboxylic groups are present in accordance with spectra of biochar made by fast pyrolysis from different types of wood [17,21,56] with transmission minima at 3393 cm^-1^ for untreated and 3198 cm^-1^ for treated SW500f. Shoulders are mutually observed for both samples close to the observed minima. For HW500f, two minima are observed for the untreated sample at 3375 cm^-1^ and 3196 cm^-1^, whereas the treated sample shows a single minimum at 3194 cm^-1^ and a shoulder at the higher wavenumber. A superimposed weak peak at 3055 cm^-1^ for SW500f and 3059 cm^-1^ for HW500f is due to the presence of aromatic CH groups [56]. The bands at 2943, 2900 and 2839 cm^-1^ for SW500f as well as 2916 and 2845 cm^-1^ for HW500f can be assigned to aliphatic CH stretching vibrations [21] in accordance with the relatively high fraction of sp^3^ hybridized carbon (Table 7). The signals seem to decrease for the treated products, but they may be interfered by the artefacts mentioned above. The characteristic bands at 1696 cm^-1^ for SW500f and 1693 cm^-1^ for HW500f are attributed to C = O stretching vibrations of carboxylic acids [56] and/or carbonyl groups [85], which are more frequent according to XPS. The presence of bands at 1580 cm^-1^ for SW500f and 1593 cm^-1^ for HW500f is due to aromatic skeletal vibrations [81]. They may contain contributions from conjugated systems such as quinones, diketones, ketoesters and keto-enol structures at the higher wavenumber side [81]. Untreated SW500f exhibits a sharp band at 1512 cm^-1^ that is also present in HW500f at 1513 cm^-1^. In treated SW500f, this band is less pronounced. It may be attributed to furan derivatives [85], which are usually present in the volatile components of fast pyrolysis biochars [91]. Bands at 1423 cm^-1^ for both biochars reflect the presence of deformation vibrations of CH_2_ and CH_3_ groups neighboring to C = C and C = O bonds [85]. The broad bands of overlapping peaks between 1400 and 1000 cm^-1^ with minima at 1202 cm^-1^ for SW500f and 1212 cm^-1^ for HW500f can be associated with ether, epoxide and phenol structures [81]. Untreated SW500f shows a strong band at 1032 cm^-1^ that disappears after treatment with HCl. This absorption is due to C-O stretching vibrations in alcohols [85]. The bands of HW500f in this spectral region are less well resolved probably due to the presence of Si-O vibrations of quartz [88] that is present in this biochar in larger amount (see Fig 3 and Table 3). The bands at 859, 802 (treated sample only) and 744 cm^-1^ for SW500f as well as 875 and 802 cm^-1^ (treated sample only) for HW500f are due to aromatic out-of-plain CH deformation. The peak at 589 cm^-1^ for untreated SW500f may stem from OH out-of-plain vibrations, but appears at very low wavenumber. It is lacking for the treated sample. The most conspicuous changes in transmission between the spectra of the treated and the untreated sample are the loss of the signals for OH groups and differences in the range between 1000 and 1500 cm^-1^ where the absorption of diverse oxygen functional groups is found. The presence of such groups is supported by the high fraction of C-O groups determined by XPS for the biochars made by fast pyrolysis. Therefore, the difference in the spectra of the HCl treated and the untreated samples is most probably due to the elution of low molecular weight components that are soluble in aqueous solution. The effect is much stronger for SW500f concerning OH stretching vibrations and not visible for HW500f concerning C-O stretching vibrations. For this char the relative intensity of the broad peak at about 1210 cm^-1^ increases after treatment with HCl, because the relative amount of SiO_2_ increases, if organic components were removed.

#### Slow pyrolysis biochar

**Fig 6:**
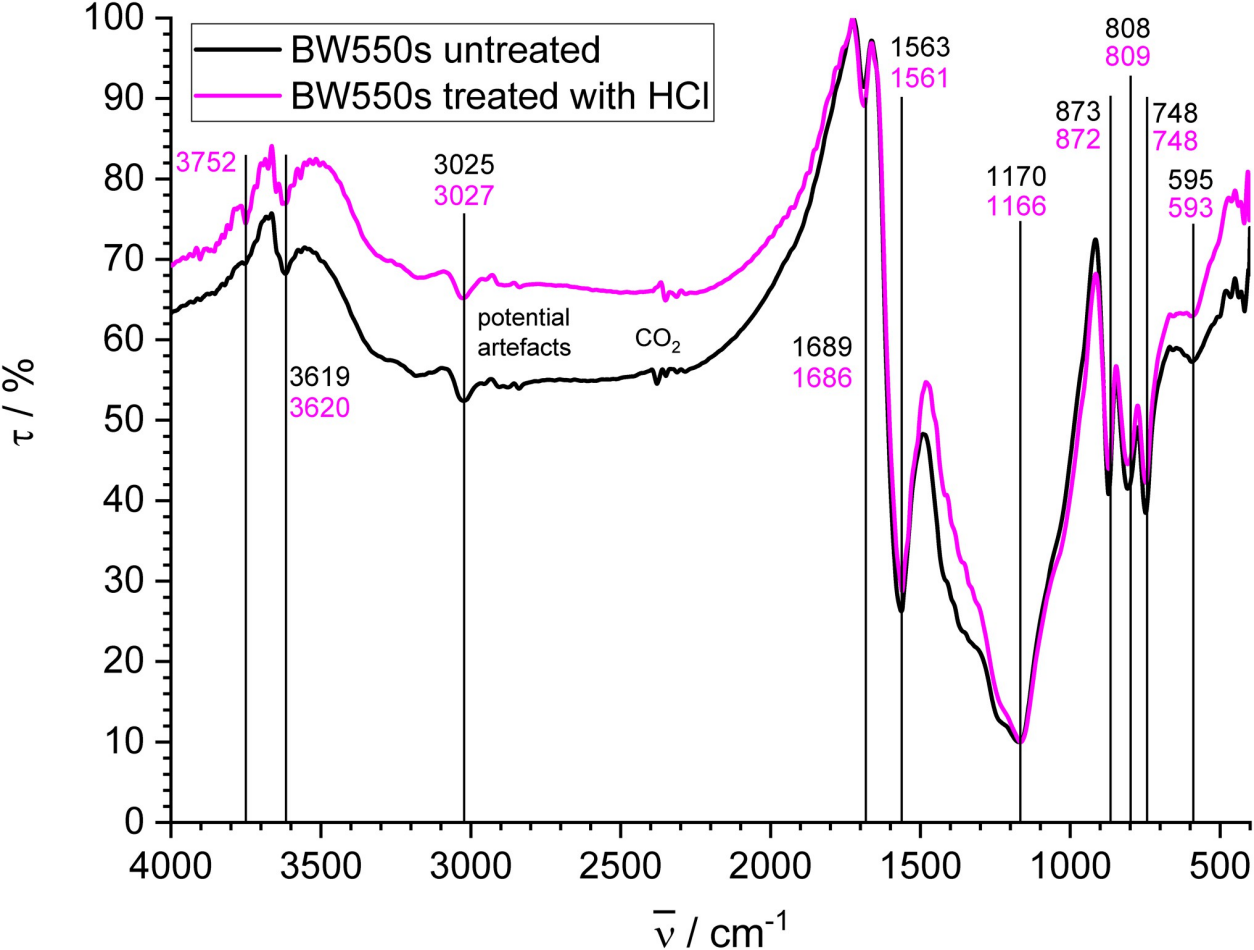
FT-IR spectra of BW550s made by slow pyrolysis (untreated and treated with HCl).

FT-IR spectra of treated and untreated slow pyrolysis biochar BW550s are given in Fig 6. The spectrum is similar to those of biochars from diverse wood made by slow pyrolysis at 400 °C within 2 h, but from much smaller feedstock particles [92] and at much smaller scale, what may influence the product [86]. The strongest and best-resolved bands of this char are typical for aromatic compounds. Some other bands are due to oxygen functional groups, which are, however, present only in relatively small amount according to element analysis and XPS. The band at 3752 cm^-1^ for the treated sample, which is also slightly visible for the untreated sample, may be attributed to free OH groups, but is out of the usual range for organic molecules [85]. The bands at 3619 (untreated) and 3620 cm^-1^ (treated) are typical for free OH groups in alcohols, phenols and carboxylic acids [85]. A broad double band with relatively low intensity is present between 3060 and 3500 cm^-1^ due to H-bonded OH stretching vibrations [86,88], which contain probably contributions of sorbed water [87,88]. The more pronounced peak at 3025 (untreated) and 3027 cm^-1^ (treated) is due to aromatic CH stretching vibrations. The intensity of aliphatic CH bands between 2800–2890 cm^-1^ is very low and peaks may be interfered by the instrumental artefacts described above, however, they should be present according to 30% sp^3^ hybridized carbon found by XPS.

The C = O stretching vibration at 1689 (untreated) and 1686 cm^-1^ (treated) can be assigned to carboxylic acids, aldehydes and ketones [93]. The band at 1563 (untreated) and 1561 cm^-1^ (treated) is due to skeletal vibrations of aromatic rings [94]. The broad band with the minimum at 1170 (untreated) and 1166 cm^-1^ (treated) is due to aromatic in-plain CH deformation vibrations [85] and may contain overlapping peaks of oxygen functional groups (few oxygen content here). The bands at 873 (untreated) and 872 (treated), 808 (untreated) and 809 (treated) and 748 cm^-1^ (treated and untreated) for aromatic out-of-plain CH deformation [85,94] are well resolved and relatively strong and reflect the relatively large H/C ratio at concomitant low O/C ratio. It also corresponds to the well-visible peak for aromatic CH stretch vibrations. The peak at 595 cm^-1^ (untreated) and 593 cm^-1^ (treated) for out-of-plain-vibrations of associated OH groups [86] corresponds to the peaks above 3000 cm^-1^. The largest relative change between the treated and the untreated sample is found in the range between 1200 and 1500 cm^-1^ and attributed to the dissolution of carbonate as will be demonstrated more clearly for the gasification biochars.

#### Gasification biochars

**Fig 7:**
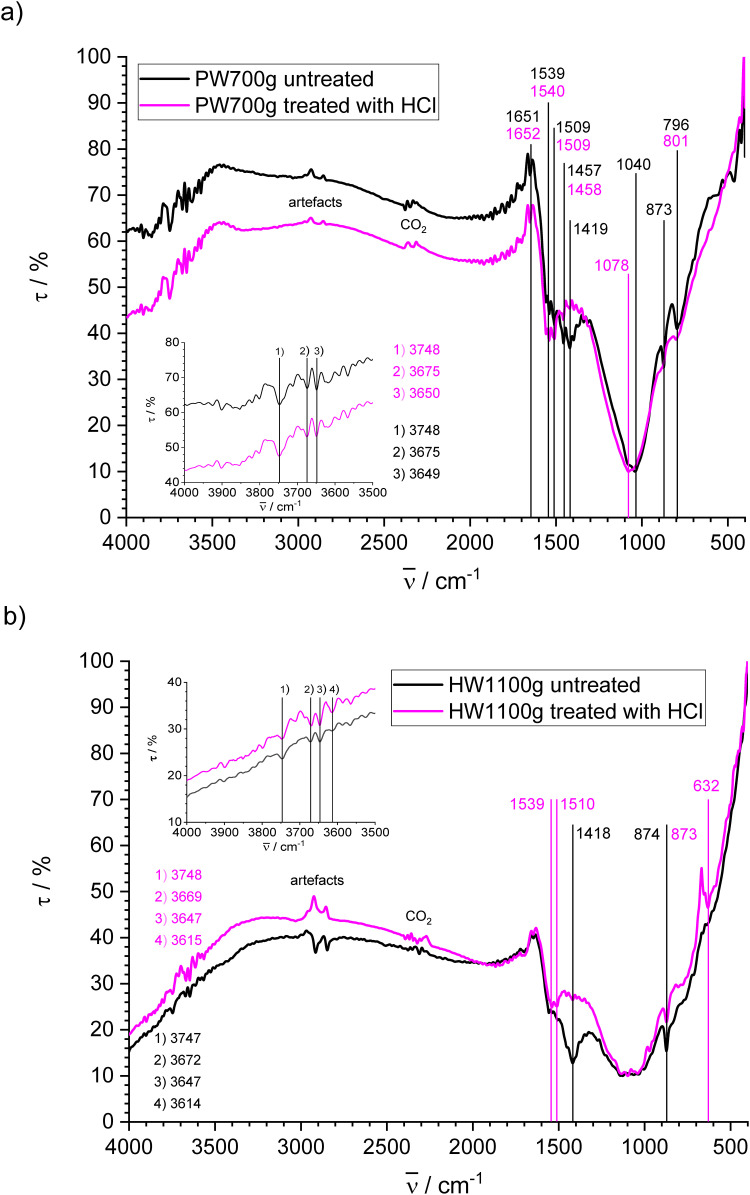
FT-IR spectra of biochars made by gasification and untreated or treated with HCl; a) PW700g; b) HW1100g.

The FT-IR spectra of gasification biochars PW700g and HW1100g treated with HCl and untreated are given in Fig 7. They are less structured than the spectra of all other chars and exhibit only very broad bands above 1000 cm^-1^ in accordance with FT-IR photoacustic spectra in the literature [56,95]. The quality of spectra is hampered by artefacts and all spectra are interfered by wiggles which may obscure weak or generate artificial peaks. Two negative bands with higher transmission at 2860 and 2915 cm^-1^ obtained from the treated samples are clearly artefacts. For the untreated samples these peaks are negative for PW700g (Fig 7a) and positive for HW1100g (Fig 7b). These relatively strong bands obscure potential weak peaks of CH stretching vibrations from aliphatic carbon that should be present in smaller amount according to XPS. Potential peaks obtained for both gasification biochars between 3500 and 3800 cm^-1^ may be assigned to stretching vibrations of free OH groups, but are interfered by wiggles and partially out of the range given by Pretsch et al. (3200–3650 cm^-1^ for free and H-bonded OH groups) [85].

The FT-IR spectra of PW700g (treated with HCl and untreated) show an expanded flat band between 3000–3500 cm^-1^. It can be attributed to OH stretching vibrations. Aromatic CH stretching vibrations cannot be distinguished from the broad OH band. There is a sharp, but weak peak at 1651 (untreated) and 1652 (treated) cm^-1^, which could be attributed to C = O stretching vibrations, but wiggles with similar intensity are nearby and therefore clear identification is not possible. The band between 1640 and 1450 cm^-1^ with transmittance minima at 1540 cm^-1^ and 1509 cm^-1^ for the HCl treated sample is mainly due to aromatic skeletal vibrations [96]. They may contain contributions from C = O and C = C olefinic moieties [85]. They are also present in the same region for the untreated product at 1539 cm^-1^ and 1509 cm^-1^, but the band is somewhat obscured by the presence of a broad band with several peaks and a minimum at 1419 cm^-1^. The very broad band between ca. 900 and 1300 cm^-1^ with minima at 1078 cm^-1^ for the treated and 1040 cm^-1^ for the untreated PW700g is assigned to CH in-plain deformation, OH deformation and C-O stretching vibrations in accordance with results from XPS. For untreated PW700g, a peak at 873 and another one at 796 cm^-1^ could be associated with CH out-of-plain deformation vibrations. A shoulder and a weak peak at 801 cm^-1^ with lower intensity compared to the large bands of the untreated sample are also observed for the HCl-treated PW700g at this positions. This result suggests that the untreated char contains aromatic components that can be washed out by HCl and explains the relatively large UV absorbance of the eluate compared with BW550s and HW1100g. The difference between the FT-IR spectra obtained from samples with and without HCl treating also confirms the presence of carbonates detected by XRD. The peak at 1419 cm^-1^ decreases after HCl treating in accordance with the spectrum of CaCO_3_ which exhibits a very strong broad band at 1415 cm^-1^ [97].

The FT-IR spectra of HW1100g show very small signals between 3500–3000 cm^-1^. There are no clear signals between 1630 and 1800 cm^-1^, which could be assigned to stretching vibrations of C = O species [81,98] in carboxylic acids and their derivatives, i.e. acids, lactones and esters due to the presence of wiggles. The band with two transmission minima at 1539 and 1510 cm^-1^ in the HCl treated HW1100g is due to aromatic skeletal vibrations [85,94]. As for PW700g, it is also present in the untreated sample, but overlaps with a broad band at 1418 cm^-1^, which disappears after treating with HCl and can be attributed to CaCO_3_ in accordance with XRD. The very broad bands between 900 and 1300 cm^-1^ for both HW1100g samples may contain overlapping CH in-plain deformation, OH deformation and C-O stretching vibrations [85]. At the flank of these bands at lower wavenumbers, peaks are present for the treated and the untreated sample which however do not completely coincide. A peak at 874 cm^-1^ for the untreated sample and at 873 cm^-1^ is present in both spectra and can be attributed to aromatic CH out-of-plain-vibrations, but a peak at 632 cm^-1^ in the treated sample, which might come from OH out-of-plain vibrations, is not present or obscured in the spectrum of the untreated material. The presence of such vibrations for the treated product seems feasible because the char is alkaline and may contain phenolate groups that are protonated when treated with HCl.

#### Hydrochars

**Fig 8:**
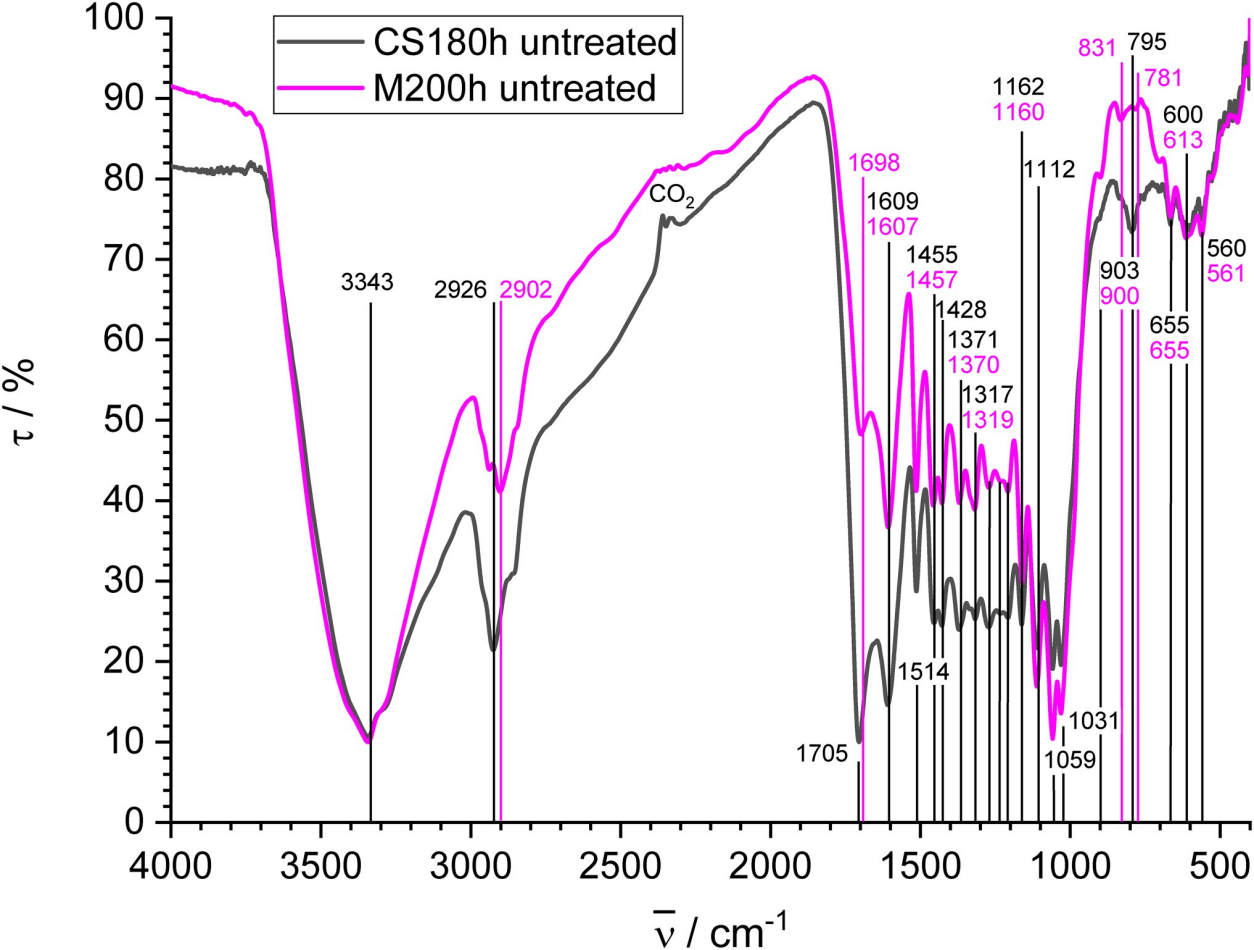
FT-IR spectra of biochars CS180h and M200h.

The FT-IR spectra of CS180h and M200h are shown in Fig 8. The spectra of both hydrochars produced from different feedstock are similar in shape, but with different intensity for some neighboring bands. They look essentially like those of mixtures of cellulose and lignin, but the bands are often shifted and broadened. It is well known for pyrochars that temperatures up to 200 °C hardly modify the chemical structure of lignocellulosic biomass [86]. The wavenumbers of the bands attributed to cellulose are very similar to values in the literature [99]. All strong bands from lignin isolated from *Miscanthus* by an Organosolv process with ethanol [100] are also present in the spectra of CS180h and M200h. They also correspond quite well with bands of bamboo that exhibits a typical spectrum of HGS type lignin from grasses (*Poaceae*) [101]. The broad band between 3700–3000 cm^-1^ with minima at 3343 cm^-1^ is attributed to OH stretching vibrations in hydroxyl or carboxyl groups in cellulose, lignin [99] and hydrochar. Intense bands at 2926 cm^-1^ for CS180h and 2902 cm^-1^ for M200h stem from CH aliphatic stretching in cellulose and lignin [99]. The broad band at 1705 cm^-1^ for CS180h and 1698 cm^-1^ for M200h which extends until 1800 cm^-1^ can be assigned to stretching vibrations of unconjugated C = O groups in lignin [99–101] and may well contain contributions of carboxyl groups (acids, esters and lactones) or carbonyl groups (quinones, ketones and aldehydes) [52] formed during hydrothermal carbonization. However, esters and lactones usually ought to be hydrolysed under the conditions of HTC. The band at 1640 cm^-1^ found and assigned to adsorbed water or conjugated C = O by Pandey et al. [99] is lacking for both hydrochars. This finding supports the interpretation as adsorbed water that may be lacking in the chars due to their reduced hydrophilicity and drying. Bands at 1609 cm^-1^ for CS180h and at 1607 cm^-1^ for M200h may be due to aromatic skeletal and C = O stretching vibrations of lignin [99], but they are somewhat out of the range given in the literature for lignin [99]. They may also contain contributions from C = C stretching vibrations in enols [85]. Bands at 1514 cm^-1^ are attributed to aromatic skeletal vibrations. They are shifted to slightly higher wavenumbers and broadened with respect to lignin, probably due to an additional contribution from carbonization. The bands at 1455 and 1457 cm^-1^ for CS180h and M200h are related to asymmetric CH_2_ and CH_3_ deformation in lignin, but appear at slightly lower wavenumbers than the limits given by Faix [101]. The band at 1428 cm^-1^ can be assigned to aromatic skeletal vibrations combined with CH in-plain deformation. It overlaps with asymmetric CH deformation from cellulose [99]. Overlapping vibrations of cellulose (symmetric CH deformation [99]) and lignin (phenolic OH in-plain deformation [99]) yield bands at 1371 (CS180h) and 1370 cm^-1^ (M200h). Bands at 1317 (CS180h) and 1319 cm^-1^ (M200h) are related to cellulose CH_2_ wagging [99]. Several bands are present between 1300 and 1200 cm^-1^, but are not explicitly mentioned with wavenumbers in Fig 8. The bands at 1274 and 1272 cm^-1^ for CS180h and M200h may be assigned to guaiacyl ring breathing and C = O in lignin, but their wavenumbers are slightly higher than the reported range [101]. Bands at 1234 cm^-1^ for CS180h and 1231 cm^-1^ for M200h are in the range for signals of aryl C-O stretch vibrations [85] but somewhat higher than values found by Faix for lignin [101]. Bands at 1208 cm^-1^ can be assigned to OH deformation in cellulose. According to Faix [101] a band at 1166 cm^-1^ is characteristic for HGS type lignins. He assigned this band to C = O in conjugated ester groups, but they may rather belong to asymmetric stretching vibrations of C-O-C in esters [85]. This band overlaps with those of C-O-C asymmetric stretching vibrations of cellulose. CS180h and M200h show corresponding bands at 1162 and 1160 cm^-1^. The band at 1112 cm^-1^ is typical for asymmetric glucose ring stretching [99]. The band at 1059 cm^-1^ is also attributed to cellulose (C-O stretching) [99] and the band at 1031 cm^-1^ is an overlapping signal of cellulose (C-O stretching) [99] and various potential vibrations of lignin [100,101]. Very weak bands at 903 and 900 cm^-1^ for CS180h and M200h are assigned to glucose ring stretching and C_1_-H deformation in cellulose [99]. The transmission minimum for M200h at 831 cm^-1^ corresponds well with aromatic CH out-of-plain deformation of lignin typically found at about 830 cm^-1^. This band is visible only as a shoulder on a broad peak situated at 795 cm^-1^ for CS180h, which is probably due to carbonized material. A much smaller peak of the same origin may be visible in the spectrum of M200h at 781 cm^-1^. Cellulose and lignin show adsorption bands in the range below 700 cm^-1^ [99] that are also present in the spectra of CS180h and M200h and may be best attributed to OH out-of-plain deformation [85]. Overall, the spectra of CS180h and M200h essentially show structural elements of unaltered or slightly decomposed cellulose and lignin, but the shift and broadening of bands and the bands at 795 and 781 cm^-1^ suggests the presence of carbonized material. Since the spectra of carbonized materials are dominated by two strong and broad signals with minima at 1550 ± 50 and 1110 ± 110 cm^-1^ bands of other origin in these ranges might be shifted. The bands originating from lignin that fall out of the ranges given in Faix [101] belong to structures that are also present in chars and therefore shifted signals are expected.

Overall, FT-IR spectra of biochars can provide valuable information on the functional groups present in biochars. They are very sensitive to the presence of OH groups, but discrimination and quantification of different types of OH groups are difficult due to the interference of water and the loss of intensity for chars made at higher temperature. Attempts to quantify different acidic functional groups by Boehm titration [57,59] yielded unreliable results for too many of the chars and are not included into this paper. Together with other analyses, FT-IR might be suitable for the quality control for chars made at lower temperature, in particular when different pretreatment is used. Extraction with diverse solvents (acids, alkaline solutions, organic solvents) could provide additional information.

### Raman spectroscopy

**Fig 9:**
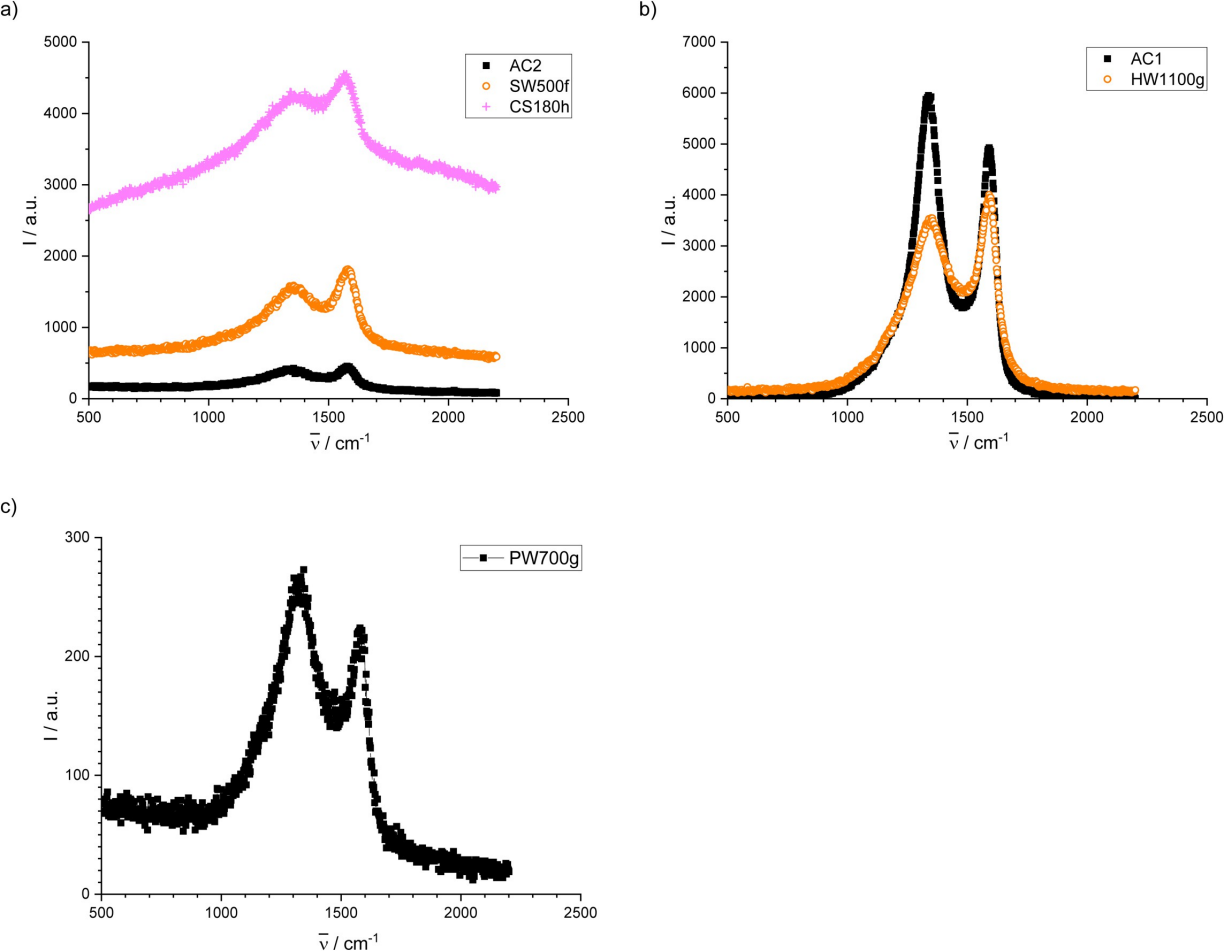
Raman spectra of biochars; a) AC2, SW500f, CS180h; b) AC1, HW1100g; c) PW700g.

Fig 9a shows the Raman spectra of AC2, SW500f and CS180h, Fig 9b the spectra of AC1 and HW1100g and Fig 9c the spectrum of PW700g. The spectra of other chars were similar to spectra shown in the figures, HW500f to SW500f, BW550s to HW1100g and M200h to CS180h. The spectra of Fig 9a exhibit low intensity and a height of the G peak that is larger than that of the D peak. The spectra in Fig 9b show higher intensity. AC1 shows a higher intensity of the D peak, whereas HW1100g shows a higher G peak. The spectrum of PW700g (Fig 9c) has very low intensity and like AC1 a higher D peak.

The intensity ratio of D and G peak (I_D_/I_G_) was determined for the spectra after a linear base line correction for the pyrochars and activated carbons by fitting 2 Lorentzian peaks to the data. The quality of the spectra of hydrochars is poor, because the D and the G peak lay on a broad signal, which is probably due to the luminescence of inorganic ions and aromatic compounds of lower molecular weight. Therefore, the baseline correction was performed by a fit of three Gaussian signals to the recorded spectrum and the subtraction of the third broad peak that can be assumed present in addition to both Raman signals of the hydrochars. After subtraction of the third peak, the spectra were fitted by 2 Lorentzian peaks. The values for the wavenumbers of the maxima (${\bar{\nu }}_{max}$), the intensity ratio I_D_/I_G_, and the in-plain crystallite size L_a_ of graphitic carbon obtained from I_D_/I_G_ according to the literature by L_a_ = (- 12.6 nm + 0.0333 λ_L_)/(I_D_/I_G_) [102,103] (with the laser wavelength λ_L_ = 532 nm) are given in Table 9.

**Table 9:** Ratio of the heights of D and G peak (ID/IG), crystallite size calculated from ID/IG, and the wavenumbers ${\overset{-}{\nu }}_{max}$ of peak maxima of the Raman spectra of the chars.

Char	I_D_/I_G_	L_a_ / nm	${\overset{-}{\nu }}_{max}$(D) /cm^-1^	${\overset{-}{\nu }}_{max}$(G) / cm^-1^
AC1	2.65	1.9	1336	1588
AC2	3.22	1.5	1337	1576
SW500f	3.12	1.6	1349	1578
HW500f	2.95	1.7	1345	1578
BW550s	2.90	1.7	1348	1587
HW1100g	3.00	1.7	1343	1587
PW700g	4.07	1.2	1323	1573
CS180h	2.01	2.5	1348	1564
M200h	2.04	2.4	1350	1563

Raman spectroscopy gives information about ordered and disordered graphitic structures of chars. Pure graphite with well ordered layers yields a single peak at 1575 cm^-1^ that is related to the E_2g_ mode of a graphite crystal of extended (infinite) size [104]. Polycrystalline graphite exhibits a second peak at 1355 cm^-1^ that is assigned to the A_1g_ mode, which becomes Raman active for finite crystal size [104]. The intensity of that peak and the peak position are related to the crystal size [104] and depend on the wavelength of the exciting light [103¸ 105]. This wavelength dependence is discussed to be due to double resonant Raman scattering [106] or coupling between electrons and phonons with the same wave vector near the K point of the Brillouin zone [103].

There are two characteristic peaks in the measured spectra of all chars, the D band with maxima between 1323 (AC1) and 1350 (SW500f) cm^-1^ assigned to disordered carbon (defects, ripples and edges), and the G band with maxima between 1563 (M200h) and 1588 cm^-1^ (HW1100g) arising from the vibration of sp^2^-bonded carbon atoms (E_2_g mode) [107]. No reliable correlation could be found for the crystallite size or the peak maxima of the series of chars from different processes neither with the temperature of production (for the biochars only), nor with the O/C or H/C ratio, nor with C-C sp^2^. Although there are contrasting results on the dependence of the crystallite size with increasing temperature for series of biochars in the literature [15,21,46,107,108], it seems not plausible that the crystallite size should be largest for the hydrochars. The spectra of both hydrochars are similar to results in the literature [52], but they do obviously not represent a suitable measure for the graphitization. The peaks may comprise contributions of components that are not carbonized. Lignin and cellulose that are still present in hydrochars according to XRD and FT-IR show Raman signals in the range between 1000 and 1700 cm^-1^ [109]. In addition, I_D_/I_G_ values for the fast pyrolysis biochars may be interfered by signals of low molecular weight components.

Overall, Raman spectroscopy seems to be not useful for determining the degree of graphitization for biochars made at lower temperature. It might be suitable for materials that are carbonized at higher temperatures [46]. The method seems also not appropriate for quality control, because the spectra show only few structure.

#### Spectral induced polarization

**Fig 10:**
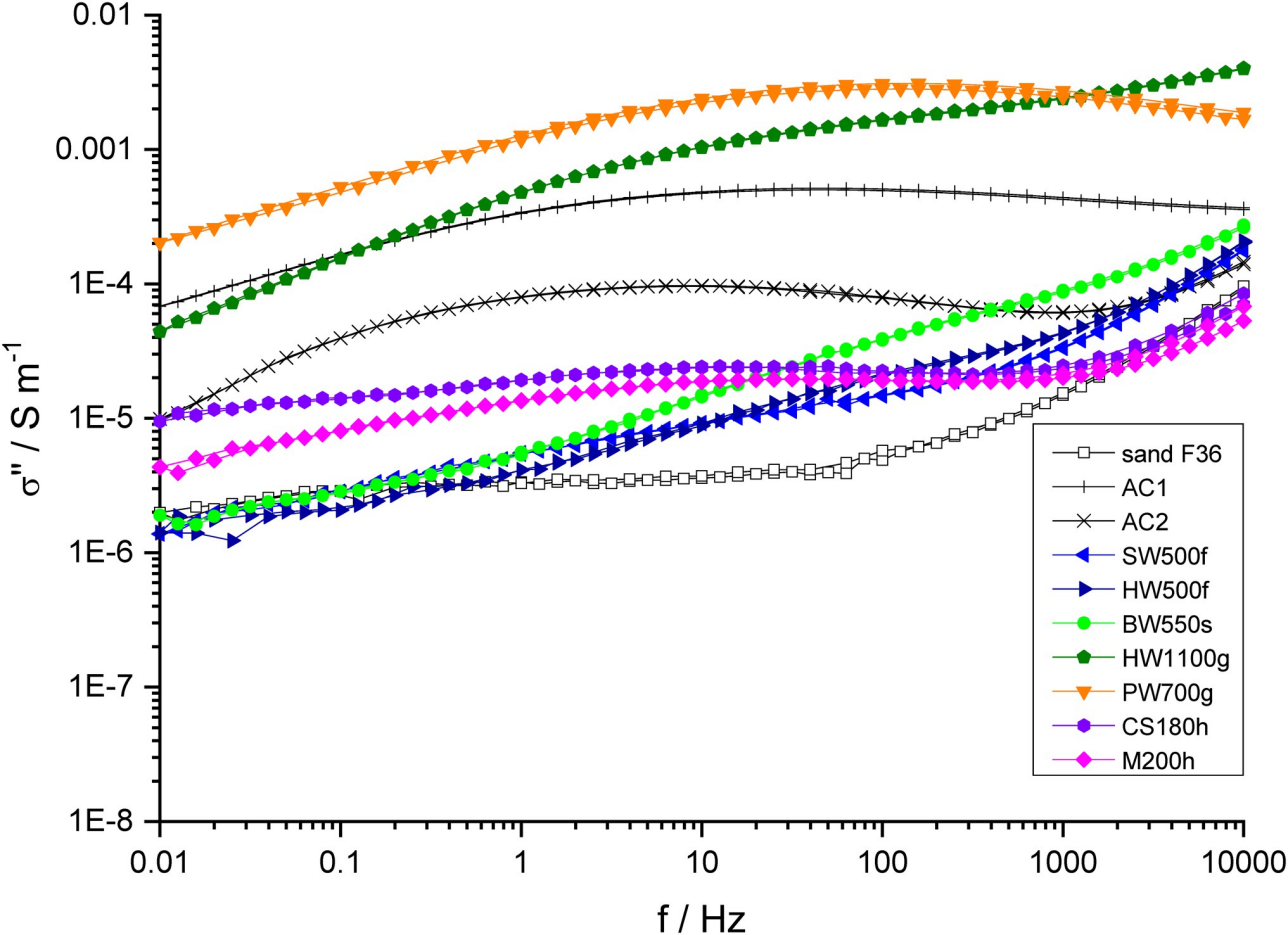
Imaginary part of the complex electrical conductivity of 2% mixtures of the chars with sand saturated with 4 mM NaCl.

Fig 10 shows the imaginary part σ" of the complex electrical conductivity of pure sand F36 saturated with 4 mM NaCl solution and mixtures of this sand with 2% (w/w) of the different chars with the same electrolyte in a double logarithmic plot in the frequency range from 10 mHz to 10 kHz. All chars show larger σ" than pure sand indicating a larger polarizability, but the values of σ" vary over several orders of magnitudes. Whereas the biochars from fast and slow pyrolysis, SW500f, HW500f and BW550s and the hydrochars, CS180h and M200h, show low values of less than 5·10^−5^ S m^-1^ below 1 kHz, both activated carbons and both gasification chars, HW1100g and PW700g exhibit larger values up to 5·10^−3^ S m^-1^. Some of them also show distinct maxima in the frequency range between 10 mHz and 1 kHz. The maxima of IP spectra can be correlated with the particle size and the height of the signal is correlated with the polarizability [65]. At frequencies above 100 Hz the presence of water in the saturated samples leads to an increase of σ", even for the sample with pure sand, due to the high dielectric constant of water. The hydrochars approach the curve of pure sand in the frequency range between 3 kHz and 10 kHz indicating that they polarize only at low frequencies whereas all other chars also polarize at high frequencies.

**Fig 11:**
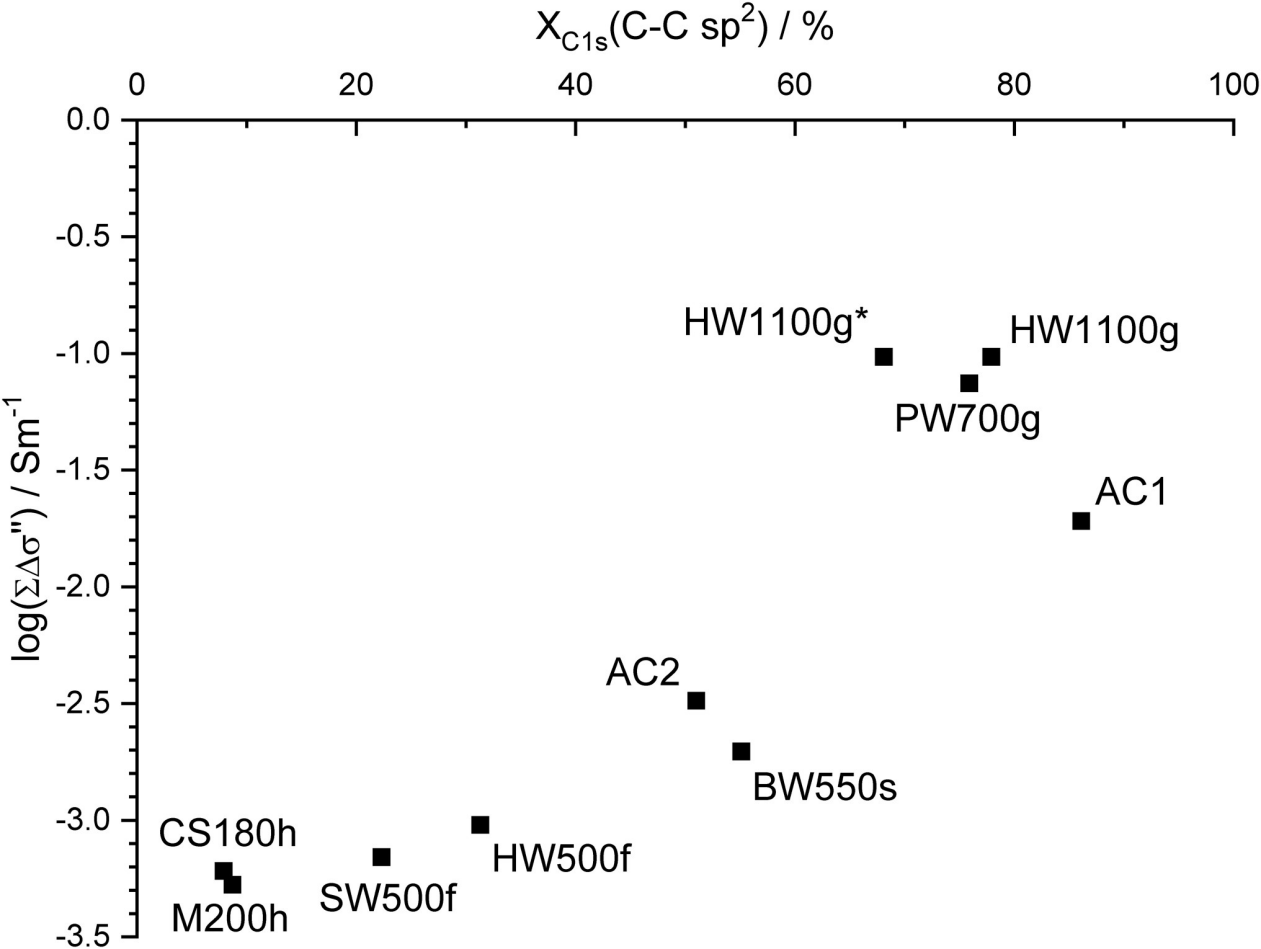
Plot of the logarithm of the sum of all measured imaginary values of the complex electrical conductivity against the fraction of sp^2^ hybridized carbon.

Fig 11 shows a plot of the integral values ΣΔσ" of the differences Δσ" between the spectra of the char samples and the sand sample over the whole spectral range from 10 mHz to 10 kHz against the fraction of sp^2^ hybridized carbon C-C sp^2^ from Table 7. There is a fair nonlinear correlation between the degree of carbonization and log ΣΔσ". The value for the polarization of AC1 is relatively low and might be a consequence of the high porosity of the sample that disturbs the free flow of electrons through the char skeleton. It is also in accordance with the high I_D_/I_G_ ratio for AC1, which suggests that this activated carbon is less graphitized than HW1100g. The high I_D_/I_G_ ratio for PW700g, however, is not in accordance with its high polarizability. A nearly identical correlation of log ΣΔσ" exists with the fraction of the π-π* satellites of the XPS spectra.

The results show that the electrical polarization of chars is very sensitive to the degree of carbonization and probably also the degree of graphitization for biochars made at higher temperatures, but the presented method is far from being routine. Modified measurements of the electrical impedance, however, could be a way for determining the degree of graphitization for carbon materials. The electrical impedance is closely related to the dielectric permittivity that has been used as a measure for graphitization by Abdelsayed et al. [15], but was determined on char powder at a single frequency in the GHz range. Such impedance or dielectric measurements are important for electrical applications of biochar anyway.

## Comprehensive discussion

The SEM images and BET values show structural similarities for each pair of chars (activated carbons, fast pyrolysis biochars, gasification biochars and hydrochars. The slow pyrolysis char is most similar to the gasification chars, but with lower specific surface area. This classification by the type of production process is found for most of the results obtained in this study, but for some methods there are considerable differences between the chars made with the same type of charring process.

Chemical analyses show a much more individual character of the chars. The differences in the van Krevelen diagram between AC1 and AC2 can be understood taking into account the presence of phosphoric acid in AC2 proven by ion chromatography. The differences in oxygen content between SW500f and HW500f as well as between HW1100g and PW700g can be explained by the extraordinarily high ash content of HW500f and PW700g due to soiling. The discrepancy between both hydrochars, however, cannot be explained by the chemical analysis. Consistent results for the sum of elements can only be obtained by determining oxygen analytically (not as the difference to 100%) and the determination of ash forming elements. This complete analysis is the base for understanding the properties of the chars. In special cases, as for the presence of phosphoric acid in AC2, additional chemical analysis might be necessary to elucidate the true composition.

X-ray diffractograms show the presence of crystalline inorganic compounds for some of the chars in accordance with the chemical analysis and a transition of the carbon-rich matrix from nearly unaltered cellulose for the hydrochars to turbostratic carbon for the gasification biochars. The results for biochars are similar to those in reference [48] but changes do not coincide with the temperature ranges given there. XRD is a necessary investigation. It yields important qualitative results on crystalline inorganic components of chars and structural properties of the char matrix, but it is not quantitative and does not reveal the presence of non-crystalline inorganic material.

Determination of the pH, the conductivity and the UV absorbance of aqueous leachates give hints on the forms of functional groups on chars, soluble inorganic matter and elutable organic material of lower molecular weight. The results support findings from other measurements and are a necessary part of quality control for the application of biochar, in particular in soil. Well elaborate extraction methods with acids, bases and organic solvents in conjunction with spectroscopic techniques might be a suitable approach for quantifying the influence of low molecular weight components on the spectroscopic properties of biochars.

XPS is a powerful method for identifying functional groups and the degree of carbonization at the surface of biochar particles, but yields only semi-quantitative results regarding the large error of the method. It is also not clear what is the relevant result for the properties of biochar. Investigations on ground HW1100g and original HW1100g* that was also milled long time ago yielded different results. The composition found at the outside of the original material might be less representative than the composition found for the ground material, because it might not be representative for the surface functional groups in the pore space. XPS can also not differentiate between alcohols and phenols and requires expensive equipment. It is therefore no method for quality control, but it is valuable for an integrated evaluation of data from other methods (chemical analysis, XRD, FT-IR, Raman and SIP). The results strongly support the classification of the investigated chars by the production method.

FT-IR spectra of hydrochars, and pyrolysis biochars exhibit a variety of bands that allow the identification of certain functional groups. Therefore, FT-IR has potential for the quality control of such biochars, but it is not a suitable method for highly carbonized materials due to the strong overlap of signals in the finger print region. When coupled with selective extraction methods, FT-IR could be quite powerful for the characterization of biochars made by pyrolysis and hydrochars, but requires elaborate protocols and the adjustment of reproducible moisture content, because OH bands are strongly affected by the presence of water. Like XPS, FT-IR cannot discriminate alcohols from phenols and therefore cannot deliver the needed information on the chemistry of surface functional groups. Titration methods that have the potential for this discrimination unfortunately failed in this study.

Raman spectroscopy that was favorably used on series of biochars [107] and coal [108] did not yield convincing results for the hydrochars and PW700g. Although the latter is a highly carbonized material according to chemical analysis, XPS and FT-IR, the Raman spectrum is poor and reveals an I_D_/I_G_ ratio that does not fit into the series of other pyrochars. The results on the hydrochars are implausible.

The electrical polarization of biochar particles might be a suitable measure for the graphitization, but other types of impedance measurements, e.g. in polymer matrices might be more suitable than SIP, which requires relatively large amount of material and relatively laborious sample preparation.

The results of this paper show that established methods used in this study have several drawbacks for several types of biochars. The most valuable results were obtained from complete chemical analysis in combination with other methods (mainly XRD, XPS, FT-IR).

The hydrochars used for this study contained a large amount of unaltered cellulose and lignin and thus exhibited properties completely different from biochars and hydrochars made at higher temperature [52]. Fast pyrolysis biochars contained a considerable amount of components of low molecular weight that contribute to FT-IR, XPS and probably Raman signals and must be distinguished from the carbon matrix. This requires pretreatment that may also change the properties of the matrix. Such changes must always be taken into account for every step of biochar handling.

## Conclusions

Although there are similarities for biochars made by the same type of process, each biochar also exhibits unique properties that must be taken into account for the choice of analytical methods that allow its full characterization and quality control. None of the spectroscopic methods used in this study was suitable for the quality control of all types of chars. Other methods like thermochemical characterization and the quantitative determination of volatile components could favorably be used for that purpose. Selective extraction methods could be suitable to separate properties of the char matrix and those coming from low molecular weight or inorganic components. A full characterization of surface functional groups is still an unresolved problem. FT-IR and XPS cannot discriminate alcohols from phenols and therefore yield only limited results. Titration methods are laborious, need sample pretreatment (changing the properties of chars) and sometimes fail in particular for chars with high ash content and reactive materials like hydrochars.

## Supporting information

S1 File Further SEM images and X-ray diffractograms with higher resolution. (PDF)
