# Supplementary material for: Surface functional groups and degree of carbonization of selected chars from different processes and feedstock
Source: PLoS One. 2022 Nov 17;17(11):e0277365. doi: 10.1371/journal.pone.0277365 (PMC9671367; doi:10.1371/journal.pone.0277365)
Supplement: S1 File — (PDF) [file pone.0277365.s001.pdf]

Supporting Information

Surface functional groups and degree of carbonisation of selected chars from different processes and feedstock

Marija Ilić[a] *, Franz-Hubert Haegel[b], Aleksandar Lolić[c], Zoran Nedić[d], Tomislav Tosti[c], Ivana Sredović Ignjatović[e], Andreas Linden[b], Nicolai D. Jablonowski[f], Heinrich Hartmann[g]

[a] University of Belgrade, Faculty of Mining and Geology,11120 Belgrade, Serbia

[b] Forschungszentrum Jülich GmbH, Institute of Bio- und Geosciences – Agrosphere (IBG-3), 52425 Jülich, Germany

[c] University of Belgrade, Faculty of Chemistry, 11158 Belgrade, Serbia

[d] University of Belgrade, Faculty of Physical Chemistry,11158 Belgrade, Serbia

[e] University of Belgrade, Faculty of Agriculture, 11080 Belgrade, Serbia

[f] Forschungszentrum Jülich GmbH, Institute of Bio- und Geosciences – Plant Sciences (IBG-2), 52425 Jülich, Germany

[g] Forschungszentrum Jülich GmbH, Central Institute for Engineering, Electronics and Analytics – Analytics (ZEA-3), 52425 Jülich, Germany

Fig. S1 shows the SEM images of selected chars not shown in the article.

Fig. S2 shows the single plots of X-ray diffractograms of all chars with higher resolution

Table S1 is a comprehension of FT-IR peaks. It is accompanied by a description.

SEM

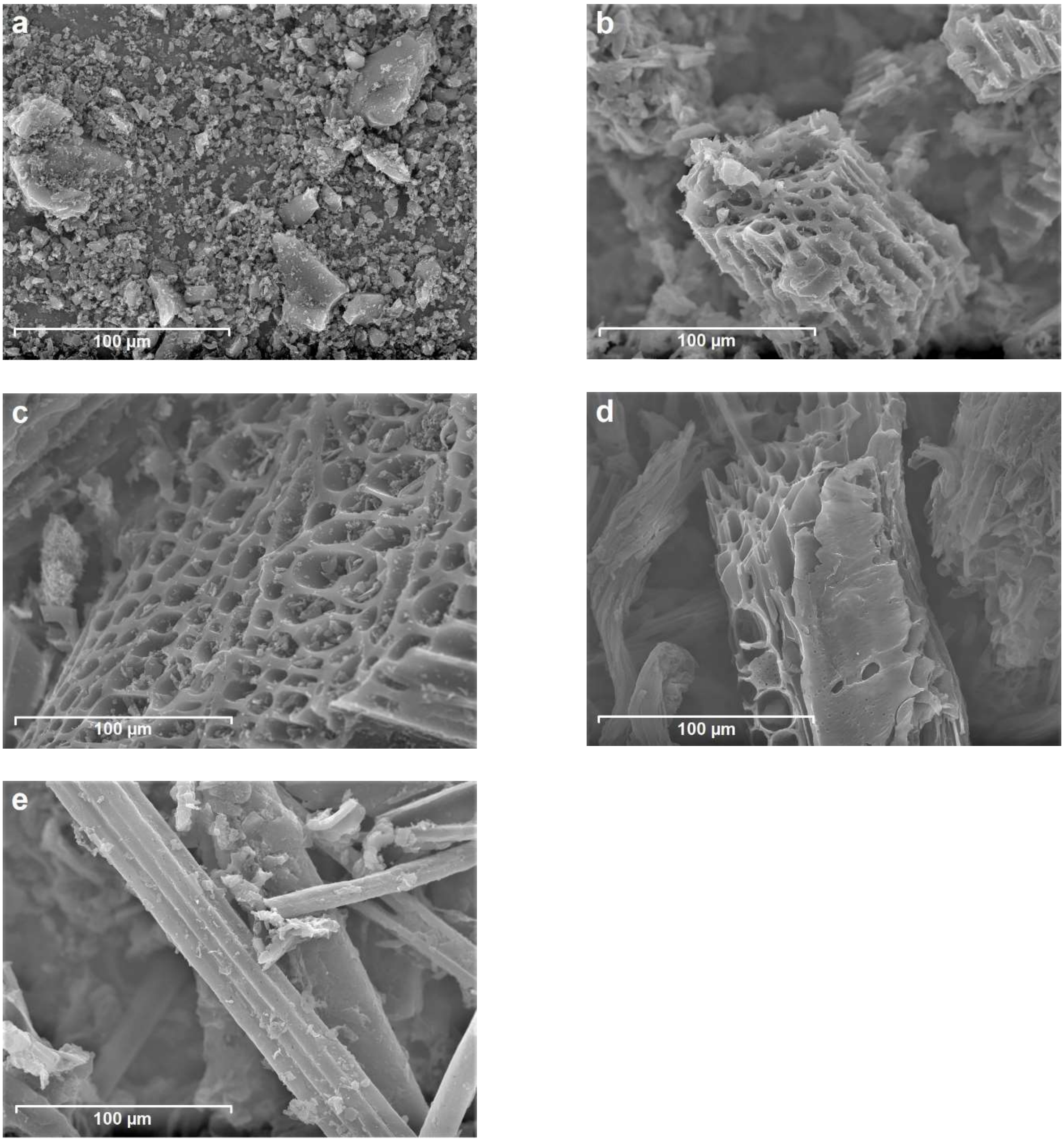

Fig. S1. SEM images of selected chars: (a) AC1, (b) BW550s, (c), HW1100g, d) HW500f and (e) CS180h.

XRD

a)

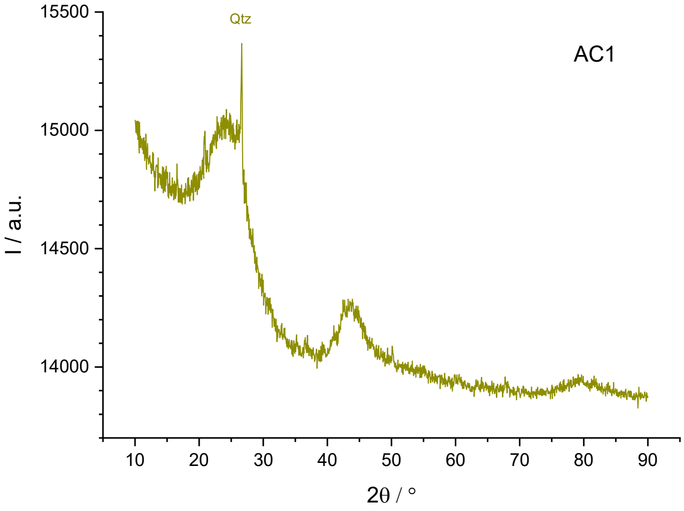

Qtz
AC1
15500
15000
14500
14000
I / a.u.
10 20 30 40 50 60 70 80 90
2θ / °

b)

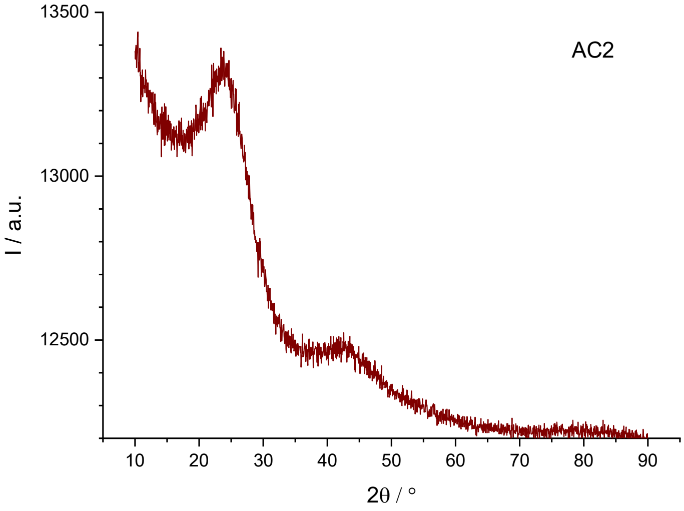

AC2
13500
13000
12500
I / a.u.
10 20 30 40 50 60 70 80 90
2θ / °

c)

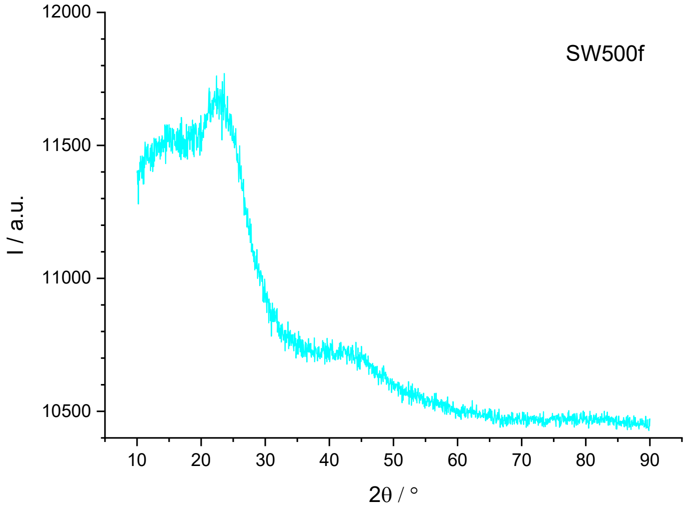

SW500f
I / a.u.
2θ / °
12000
11500
11000
10500
10
20
30
40
50
60
70
80
90

d)

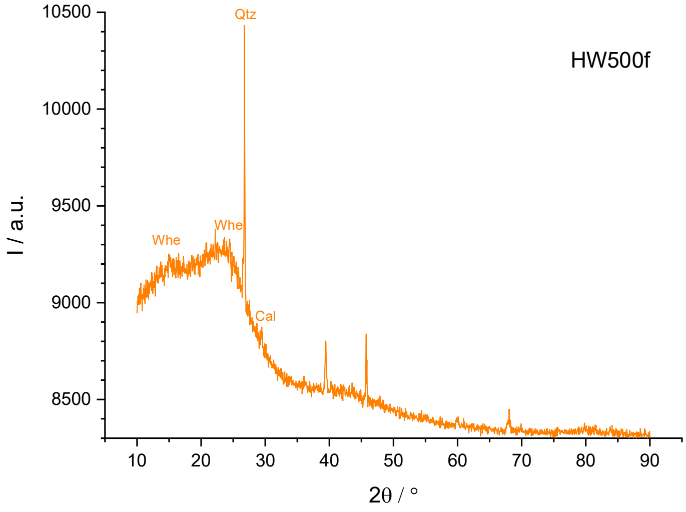

HW500f
Qtz
Whe
Whe
Cal
I / a.u.
2θ / °
10500
10000
9500
9000
8500
10
20
30
40
50
60
70
80
90

e)

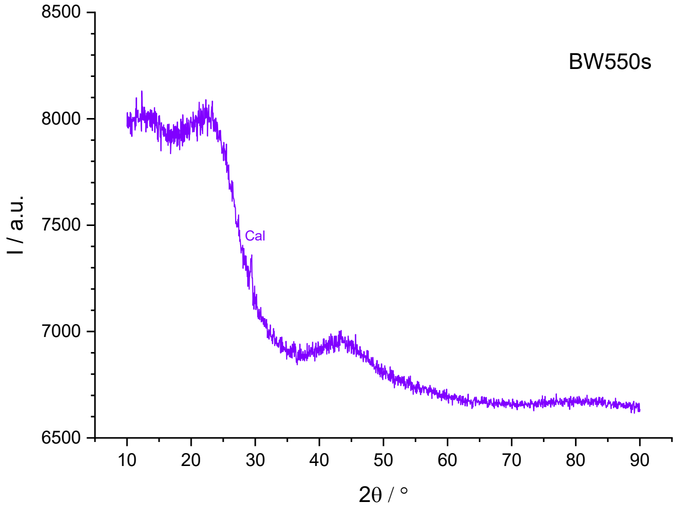
BW550s
I / a.u.
8500
8000
7500
7000
6500
Cal
10
20
30
40
50
60
70
80
90
2θ / °

f)

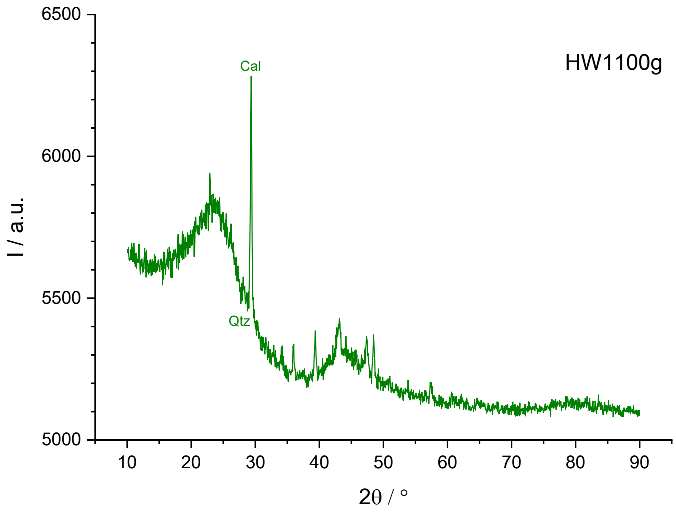
HW1100g
I / a.u.
6500
6000
5500
5000
Cal
Qtz
10
20
30
40
50
60
70
80
90
2θ / °

g)

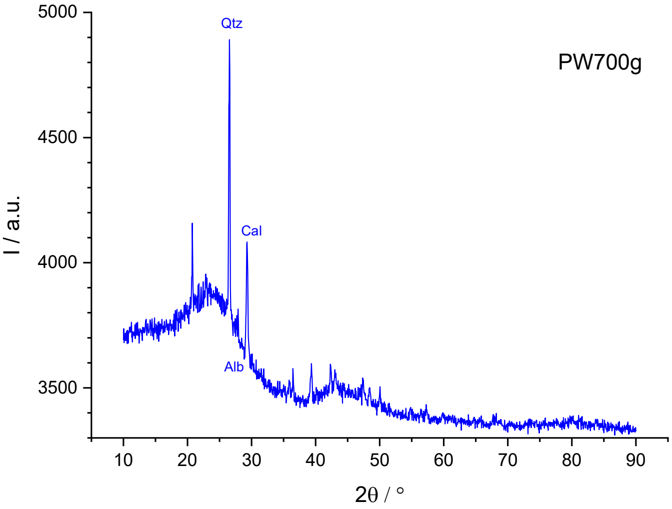

h)

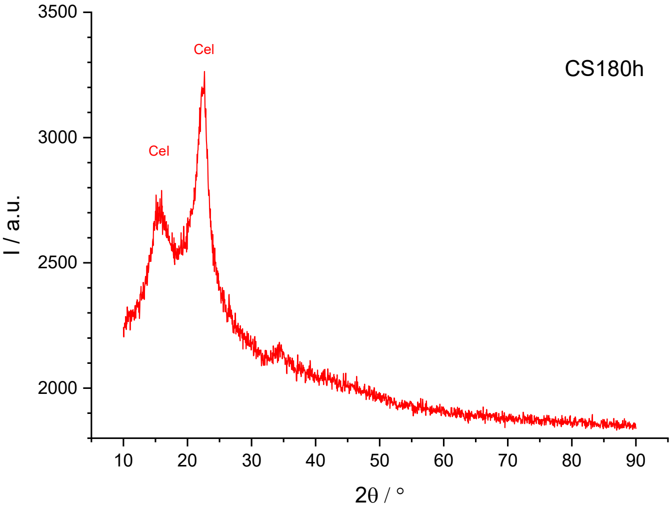

i)

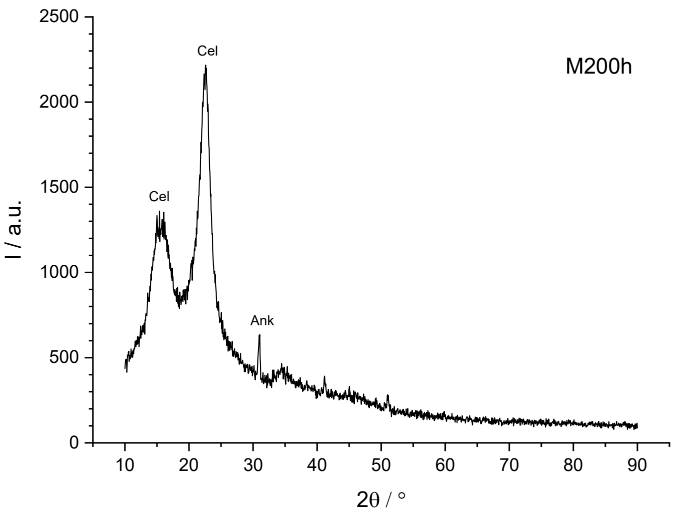

Fig. S2. X-ray diffractograms of the chars, .a) AC1, b) AC2, c) SW500f, d) HW500f, e) BW550s, f) HW1100g, g) PW700g, h) CS180h, i) M200h

FT-IR

Table S1 shows the wavenumbers for all peaks discussed in the main paper. Biochars made by slow pyrolysis and gasification showed several resolved peaks for free OH stretching vibrations, while all other chars showed broad absorption bands for associated OH stretching vibrations. Aromatic CH stretching vibrations could be identified for AC2, SW500f, HW500f, HW500f (HCl), BW550s and BW550s (HCl). They might be hidden by the large OH signal for SW500f (HCl) and both hydrochars. They are essentially lacking for AC1 and both chars made by gasification. The corresponding aromatic CH out-of-plain deformation vibrations below 1000 cm^{-1} are also lacking for AC1 and present only with minor intensity for the gasification biochars and the hydrochars. Aliphatic CH stretching vibrations were found for AC2, the fast pyrolysis biochars and the hydrochars. Bands for C=O stretching vibrations were found for all chars except AC1 where it is essentially lacking and HW1100g where they might be part of the broad band found for this char at 1418 cm^{-1} for the untreated material and the broad double band at 1539 and 1510 cm^{-1} for the HCl-treated material. These broad bands

from aromatic skeletal vibrations may obscure many peaks in the fingerprint region belonging to surface functional groups. Therefore lacking values for some functional groups in the table give the false impression that such functional groups may not be present in HW1100g. The same problem exists for the broad band between 1300 and 900 cm^{-1} and for corresponding broad bands of BW550s, PW700g, AC1 and AC2. For the less carbonized products (biochars from fast pyrolysis and hydrochars), the intensity of the bands of oxygen surface functional groups is relatively higher and some of the bands can be assigned to specific vibrations of conjugated carbonyl compounds, phenols, alcohols and ethers. Whereas the spectra of fast pyrolysis biochars show also broad broads with probably overlapping peaks, most bands of hydrochars could be assigned to vibrations of cellulose and lignin indicating that these chars were only weakly carbonized. The bands found for the fast pyrolysis biochars are partially due to material of low molecular weight as can be seen from the intensity differences in Fig. 5a and 5b between the spectra of the untreated and the HCl-treated materials. Signals at 1418 and 1419 cm^{-1} for untreated HW1100g and PW700g can be unequivocally assigned to carbonate vibrations. They are not present after the treatment with HCl. Bands from quartz should be present in the spectra of AC1, HW500f, HW1100g and PW700g in the range between 1200 and 1000 cm^{-1} but are not resolved. PO vibrations are found for AC2, the carbon that was activated with phosphoric acid. Signals below 700 cm^{-1} were assigned to O-H out-of-plain vibrations and were particularly prominent for the hydrochars.

Table S1. Peaks of FT-IR spectroscopy

Assignment / Char	AC1	AC2	SW500f	SW500f (HCl)	HW500f	HW500f (HCl)	BW550s	BW550s (HCl)	HW1100g	HW1100g (HCl)	PW700g	PW700g (HCl)	CS180h	M200h
O-H stretching free							3619	3752<br>3620	3747<br>3672<br>3647<br>3614	3748<br>3669<br>3647<br>3615	3748<br>3675<br>3649	3748<br>3675<br>3650		
O-H stretching associated	3412	3373<br>3197	3393	3198	3375<br>3196	3194							3343	3343
aromatic C-H stretching		3044	3055		3059	3066	3025	3027						
aliphatic C-H stretching		2949<br>2914<br>2845	2943<br>2900<br>2839	2914<br>2840	2916<br>2845	2914<br>2840							2926	2902
C=O stretching		1693	1696	1694	1693	1694	1689	1686			1651	1652	1705<br>1609	1698<br>1607
aromatic skeletal C=C vibration	1568<br>1544	1576	1580	1590	1593	1590	1563	1561		1539<br>1510	1539<br>1509<br>1457	1540<br>1509<br>1458	1609<br>1514	1607<br>1514
conjugated systems (C=O and C=C stretching)			1512	1512	1513	1512								

Table S1. (continuation)

Assignment / Char	AC1	AC2	SW500f	SW500f (HCl)	HW500f	HW500f (HCl)	BW550s	BW550s (HCl)	HW1100g	HW1100g (HCl)	PW700g	PW700g (HCl)	CS180h	M200h
CH_2 and CH_3 asymmetric deformation													1455	1457
aromatic skeletal vibration; C-H in-plain deformation													1428	1428
CO stretching vibration from carbonates									1418		1419			
CH_2, CH_3 deformation			1423	1424	1423	1424								
symetric C-H deformation; phenolic O-H in-plain deformation													1371	1370
CH_2 wagging													1317	1319
guaiacyl ring breathing and C=O in lignin													1274	1272
aryl C-O stretching													1234	1231
C-OH, C-O-C stretching		1193	1202	1209	1212	1209							1208	1208

Table S1. (continuation)

Assignment / Char	AC1	AC2	SW500f	SW500f (HCl)	HW500f	HW500f (HCl)	BW550s	BW550s (HCl)	HW1100g	HW1100g (HCl)	PW700g	PW700g (HCl)	CS180h	M200h
aromatic C-H in-plain deformation							1170	1166						
C-O-C stretching													1162	1160
glucose ring asymetric stretching													1112	1112
C-O, O-H and C-H stretching	1084	1081	1032								1040	1078	1059<br>1031	1059<br>1031
P-O stretching		1081												
glucose ring streching, C1-H deformation													903	900
aromatic C-H out-of-plain deformation		993<br>877<br>827<br>750	859<br>744	869<br>802	875	869<br>802	873<br>808<br>748	872<br>809<br>748	874	873	873<br>796	801	795	831<br>781
P-O deformation		494												
O-H out-of-plain vibration			589				595	593		632			655<br>600<br>560	655<br>613<br>561
